# Development of an Item Bank to Measure Medication Adherence: Systematic Review

**DOI:** 10.2196/19089

**Published:** 2020-10-08

**Authors:** Yu Heng Kwan, Livia Jia Yi Oo, Dionne Hui Fang Loh, Jie Kie Phang, Si Dun Weng, Dan V Blalock, Eng Hui Chew, Kai Zhen Yap, Corrinne Yong Koon Tan, Sungwon Yoon, Warren Fong, Truls Østbye, Lian Leng Low, Hayden Barry Bosworth, Julian Thumboo

**Affiliations:** 1 Program in Health Services and Systems Research Duke-NUS Medical School Singapore Singapore; 2 Department of Pharmacy National University of Singapore Singapore Singapore; 3 PULSES Centre Grant SingHealth Regional Health System Singapore Singapore; 4 Department of Rheumatology and Immunology Singapore General Hospital Singapore Singapore; 5 Center of Innovation to Accelerate Discovery and Practice Transformation (ADAPT) Durham Veterans Affairs Health Care System Durham, NC United States; 6 Department of Psychiatry and Behavioral Sciences Duke University School of Medicine Durham, NC United States; 7 Pharmacy Transformation Office National Healthcare Group Pharmacy Singapore Singapore; 8 Duke-NUS Medical School Singapore Singapore; 9 NUS Yong Loo Lin School of Medicine National University of Singapore Singapore Singapore; 10 Department of Family Medicine and Continuing Care Singapore General Hospital Singapore Singapore; 11 Post Acute and Continuing Care Outram Community Hospital Singapore Singapore; 12 School of Nursing Duke University Medical Center Durham, NC United States; 13 Department of Population Health Sciences Duke University Medical Center Durham, NC United States

**Keywords:** systematic review, patient-reported outcome measures, item bank, adherence

## Abstract

**Background:**

Medication adherence is important in managing the progression of chronic diseases. A promising approach to reduce cognitive burden when measuring medication adherence lies in the use of computer‐adaptive tests (CATs) or in the development of shorter patient-reported outcome measures (PROMs). However, the lack of an item bank currently hampers this progress.

**Objective:**

We aim to develop an item bank to measure general medication adherence.

**Methods:**

Using the preferred reporting items for systematic review and meta-analysis (PRISMA), articles published before October 2019 were retrieved from PubMed, Embase, CINAHL, the Cochrane Library, and Web of Science. Items from existing PROMs were classified and selected (“binned” and “winnowed”) according to standards published by the Patient-Reported Outcomes Measurement Information System (PROMIS) Cooperative Group.

**Results:**

A total of 126 unique PROMs were identified from 213 studies in 48 countries. Items from the literature review (47 PROMs with 579 items for which permission has been obtained) underwent binning and winnowing. This resulted in 421 candidate items (77 extent of adherence and 344 reasons for adherence).

**Conclusions:**

We developed an item bank for measuring general medication adherence using items from validated PROMs. This will allow researchers to create new PROMs from selected items and provide the foundation to develop CATs.

## Introduction

Medication adherence is defined as the degree to which a patient's behavior corresponds with the agreed recommendations from a health care provider [1]. The average adherence rate ranges from 50% among patients suffering from chronic diseases in developed countries [2] to 79% among those receiving medical treatment prescribed by a nonpsychiatrist physician [3,4]. Nonetheless, medication nonadherence is recognized as a significant public health issue since it can result in poor health outcomes and increased health care costs [5]. Medication adherence, which is important in managing the progression of chronic diseases, may be assessed using patient-reported outcome measures (PROMs). Using PROMs to measure medication adherence may be susceptible to a social desirability bias [6]; however, PROMs are much more practical in daily clinical practice because of their relatively low cost and ease of administration as compared to pill counting or an electronic monitoring system such as the medication event monitoring system (MEMS).

PROMs are measures of the status of a patient's health condition that originate directly from the patient, without interpretation of the patient's response by a caregiver or physician [7]. Numerous PROMs have been developed and validated to measure medication adherence. These include instruments such as the Medication Adherence Report Scale (MARS) [8], the 4- and 8-item Morisky Medication Adherence Scale (MMAS-4 and MMAS-8) [9], the Hill-Bone Medication Adherence (HBMA) scale [10], and the Domains of Subjective Extent of Nonadherence (DOSE-Nonadherence) scale [11]. However, most self-report measures that were developed using classical test theory [12,13] are static and administered using a common item set regardless of the respondent's level of medication adherence [14]. Since patients are asked the same questions repeatedly, this approach results in significant cognitive burden [15], low precision [16], a waste of the patients' time, as well as a lack of additional, new information [17].

A novel approach to overcome this limitation lies in the use of a computer‐adaptive test (CAT) to create new PROMs for measuring medication adherence. A CAT is a system for tailoring a test, whereby the next item administered to the respondent is determined by and adaptive to the patient's response to the previously-administered item [18]. The Patient-Reported Outcomes Measurement Information System (PROMIS) was developed through a CAT and item response theory (IRT). Instead of focusing on the entire test, IRT shifts the focus to the individual questions [19]. The use of IRT with a CAT allows for the identification, individualization, and administration of a feasible number of items that are likely to offer the highest precision [20]. In order to achieve higher precision, PROMIS investigators were required to identify and develop items covering the full range of experience in the domains the instrument was intended to measure (ie, content validity) [14]. Thus, the first step to a CAT is an item bank consisting of questions from medication adherence PROMs. Therefore, this systematic literature review aims to identify and develop an item bank through a comprehensive summary of the questions from validated medication adherence PROMs.

## Methods

This systematic review was guided by the preferred reporting items for systematic review and meta-analysis (PRISMA) statement [21]; standards published by the PROMIS committee [22] were adapted in the development of the item bank.

### Search Strategy

Articles published before October 2019 were retrieved from PubMed, Embase, CINAHL, the Cochrane Library, and Web of Science. A search strategy (Multimedia Appendix 1) of 4 components was used as follows: construct of interest, population, instrument, and measurement properties. The searches focused on medication adherence PROMs. Where available, the sensitivity of the searches was enhanced using search filters developed by Terwee et al [23], which involves a combination of search terms designed to retrieve studies on measurement properties of measurement instruments. The search records were downloaded into Endnote X9 (Clarivate Analytics), and any duplicates were removed.

### Article Selection

All titles and abstracts were screened independently by 2 reviewers (LJYO and SDW). A third reviewer (YHK) was consulted when a disagreement arose between the 2 reviewers. For articles that were potentially relevant, the full text of these articles was independently reviewed by the same 2 reviewers for inclusion or exclusion.

Articles were included if they were full-text original publications in English that validated PROMs for medication adherence. Articles were excluded if the PROMs were completed by proxy, or if they were conference abstracts, expert opinions, narrative reviews, or not peer-reviewed. Animal and case studies, as well as non-English language studies, were also excluded. These exclusions were not used to construct the search strategy to avoid the omission of relevant articles.

### Data Extraction

Where available, 2 reviewers (LJYO and SDW) extracted study population characteristics (sample size, age, gender, and country) data from the articles.

### Identification of Existing PROMs for Inclusion

The names of PROMs extracted from the previous step were consolidated. In order to optimize the number of relevant items for evaluation, the most recent and exhaustive version was included when multiple versions of the same PROM were found across the included studies.

For the assessment of PROMs for inclusion, reviewers obtained information regarding the PROM through internet searches. Copies of the shortlisted PROMs were retrieved either from sources available to the public (ie, official websites or research publications) or by requesting copies from the developers or study investigators of these PROMs. Permission was obtained from the study investigators for the inclusion of the PROM into the item bank. Where possible, permission from the PROM developers was sought in the case that the study investigators were not in a position to provide consent due to claims of intellectual property. After the initial contact, 2 follow-up reminder emails were sent to the unresponsive study investigators. This resulted in a final list of PROMs from which items were extracted and evaluated.

### Item Classification (Binning)

Item classification, or binning, refers to a systematic process for grouping items according to meaning and specific latent construct. This process aims to obtain a bin with the most exhaustive list of items, from which a smaller number of items may be chosen to adequately represent the bin. The number of items that would adequately represent a bin was not predetermined, as the purpose of this process was to identify sufficient items that encompass the meaning of the bin and to eliminate unnecessary redundancy in the pool of items [14].

Binning was done using terms in English, and each item was included in as many bins as was deemed fit. To ensure that the binning process was exhaustive, 2 independent reviewers (LJYO and DHFL) evaluated any one item for possible inclusion, and an item identified for inclusion to a bin by at least 1 reviewer was included in that bin.

A 2-stage process was carried out for binning. First-order binning was completed at the level of the domain: each item was evaluated for possible inclusion into (1) Extent of Adherence and/or (2) Reasons for Nonadherence. The domains were derived from the self-report measure developed by Voils et al [11].

For the Reasons for Nonadherence domain, second-order binning was completed at the level of the subdomain consisting of (1) social- and economic-related factors, (2) health care team and system-related factors, (3) therapy-related factors, (4) condition-related factors, and (5) patient-related factors. The subdomains were derived from the World Health Organisation (WHO) framework for medication adherence [2]. The framework has been widely used in various literature [24-32]. Second-order binning was not deemed to be necessary for the Extent of Adherence domain.

### Item Selection (Winnowing)

The process of winnowing aims to narrow the large pool of items down to a representative set of items; this is done by identifying item characteristics that would either include or exclude items from the item bank based on the definition of the domains [14]. Winnowing was performed by 2 reviewers (LJYO and DHFL) independently assessing each bin, and items that best represented the respective domains were first selected. The process was carried out separately for each domain.

The following criteria were used to eliminate items from consideration [14]: (1) the content of the item was inconsistent with the definition of medication adherence, or with the scope of the extent of and reasons for medication adherence; (2) the item was semantically redundant with a previous item; (3) the content of the item was too narrow to be universally applicable; (4) the stem of the item was highly disease-specific, which reduces overall applicability and limits the adaptation of the item; (5) the item was confusing; and (6) the item was open-ended, which increases the difficulty of implementation.

After the pair of reviewers completed the item selection independently, a third reviewer (YHK), who is trained in measurement science and item banking and was not previously involved in the binning and winnowing process, was consulted to identify the items that best represented each domain, as well as the items for removal.

## Results

### Search Results and Characteristics of the Included Articles

**Figure 1:**
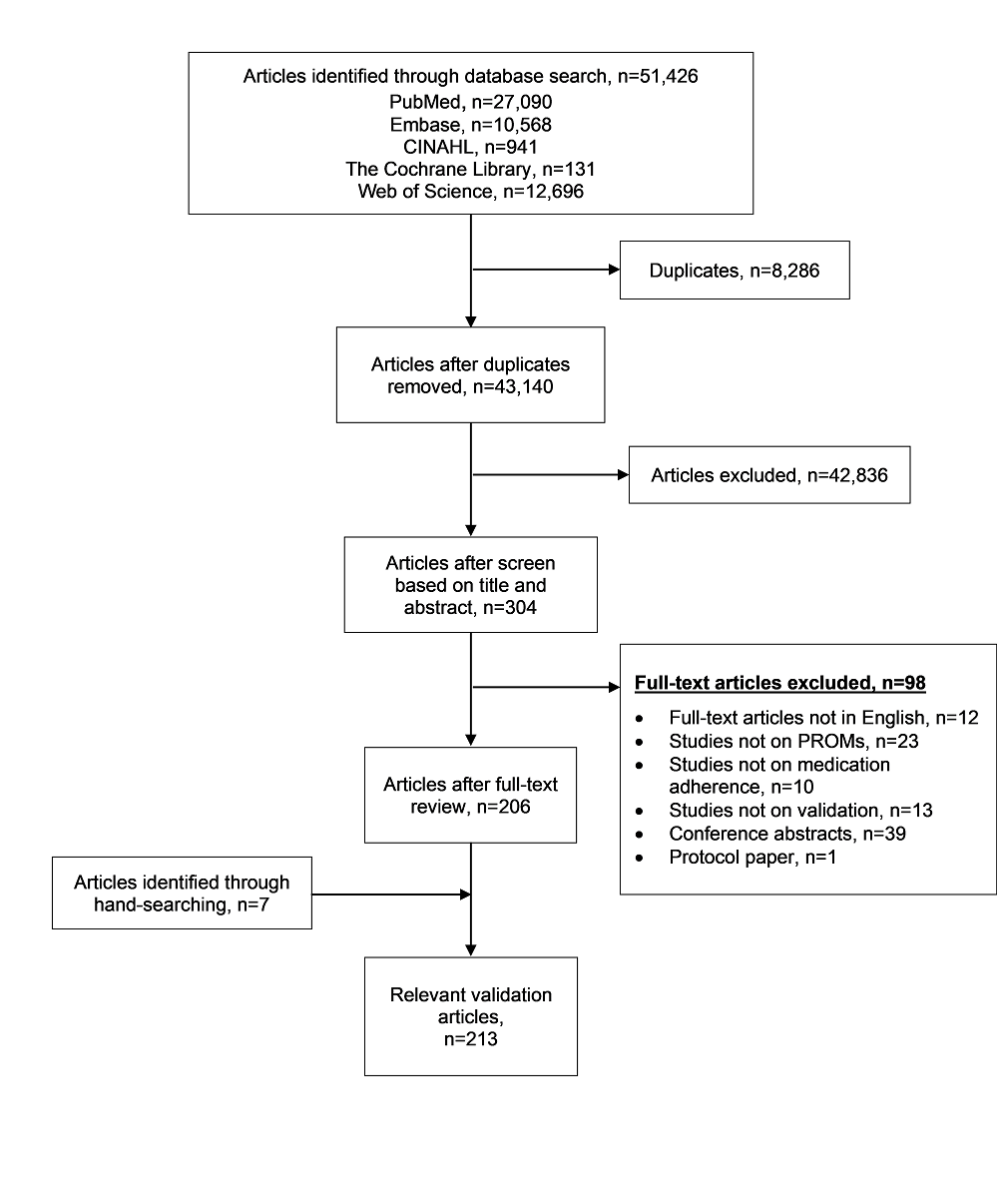
Flow chart of the systematic literature review.

A total of 51,426 studies were obtained from the database search, of which 8286 duplicates were excluded. A review of the titles and abstracts led to the exclusion of 42,836 studies. Subsequently, full-text review excluded 98 studies, with reasons provided in Figure 1. An additional 7 studies were identified through hand-searching of reference lists, resulting in 213 relevant validation studies for potential inclusion into the item bank. The characteristics of the relevant studies are presented in Table 1.

**Table 1:** Characteristics of studies for potential inclusion into the item bank (n=213).

General Characteristics		Values
Number of unique countries involved		48
Number of unique PROMs^a^ studied		126
**Sample size, n (%)**		
	< 50	17 (8.0)
	50 – 99	31 (14.6)
	100 – 199	64 (30.0)
	200 – 299	30 (14.1)
	300 – 399	22 (10.3)
	400 – 499	15 (7.0)
	> 500	34 (16.0)
**Mean age in years, n (%)**		
	0 < mean age ≤ 20	9 (4.2)
	20 < mean age ≤ 40	28 (13.2)
	40 < mean age ≤ 60	101 (47.4)
	60 < mean age ≤ 80	54 (25.4)
	≥ 80	2 (0.9)
	Not reported ^b^	19 (8.9)
**Proportion of males, n (%)**		
	< 0.2	15 (7.0)
	0.2 ≤ x < 0.4	46 (21.6)
	0.4 ≤ x < 0.6	77 (36.2)
	0.6 ≤ x < 0.8	40 (18.8)
	≥ 0.8	23 (10.8)
	Not reported	12 (5.6)

^a^ PROMs: patient-reported outcome measures.

^b^ Includes values in the form of median, range, or not reported.

### Identification of Existing PROMs for Inclusion

The review of the included articles identified 126 unique PROMs measuring medication adherence, which were validated in 48 countries. A majority of the identified PROMs were self-administered questionnaires.

After obtaining written permission from the study investigators and PROM developers for the use of the items as part of the item bank, 47 PROMs from 53 studies were included in the item bank, as presented in Textbox 1. PROMs from the remaining 160 studies (Multimedia Appendix 2 [33-189]) were excluded due to a lack of consent. Among the 53 included studies, the Modified Drug Adherence Work-Up (M-DRAW) tool, the Reduced Glaucoma Treatment Compliance Assessment Tool (GTCAT), and the 3-Item Self-Report Measure for Medication Adherence were each evaluated in 2 studies; the General Medication Adherence Scale (GMAS) was evaluated in 3 studies; and DOSE-Nonadherence was evaluated in 4 studies. PROMs included were mostly developed and validated in English. A total of 579 items were collated from these PROMs, including 71 non-English items.

List of the patient-reported outcome measures (PROMs) that were included in the item bank. *Instruments which were not named; ** instruments developed/validated in non-English but have an existing English-translated version for publication purposes; ***instruments developed and/or validated in non-English.

**Generic PROMs**

- Diagnostic Adherence to Medication Scale (DAMS) [190] (6 items)
- 7-Item Adherence to Refills and Medications Scale (ARMS-7) [191]** (7 items)
- Adherence to Refills and Medications Scale (ARMS) [192] (12 items)
- Brief Medication Adherence Scale (BMAS) [193]** (10 items)
- Domains of Subjective Extent of Nonadherence (DOSE-Nonadherence) Scale [11,194-196] (21 items)
- Every Visit Adherence Questionnaire [197] (1 item)
- General Medication Adherence Scale (GMAS) [198-200] (11 items)
- Medication Adherence Estimation and Differentiation Scale (MEDS) [201] (16 items)
- Modified Drug Adherence Work-Up (M-DRAW) Tool [202,203] (14 items)
- Self-Reported Adherence (SERAD) Questionnaire [204]*** (2 items)
- Simplified Medication Adherence Questionnaire (SMAQ) [205] (6 items)
- 3-Item Self-Report Measure for Medication Adherence [206,207]* (3 items)

**Disease-Specific PROMs**

- 12-Item Medication Adherence Scale for Patients with Chronic Disease [208]* (12 items)
- 5-Item Compliance Questionnaire for Rheumatology (CQR5) [209] (5 items)
- Adult Asthma Adherence Questionnaire (AAAQ) [210] (5 items)
- Antidepressant Adherence Scale (AAS) [211] (4 items)
- Antipsychotic Medication Beliefs and Attitudes Scale (AMBAS) [212] (12 items)
- Assessment Scale for Treatment Compliance in Type 2 Diabetes Mellitus [213]* (30 items)
- Brief Evaluation of Medication Influences and Beliefs (BEMIB) [214] (8 items)
- Chinese and Western Medication Adherence Scale in Chronic Kidney Disease [215]* (18 items)
- Chinese Diabetes Medication Self-Efficacy Scale (CDMSS) [216]*** (19 items)
- Combination Antiretroviral Therapy Adherence Questionnaire [217]* (4 items)
- Diabetes Management Questionnaire (DMQ) [218] (21 items)
- Diabetes Medication Self-Efficacy Scale (DMSS) [219] (19 items)
- ICAMP Adherence Questionnaire [220] (10 items)
- Immunosuppressant Therapy Adherence Scale (ITAS) [221]*** (4 items)
- Iraqi Anti-Diabetic Medication Adherence Scale (IADMAS) [222] (8 items)
- IRT-30 [223] (10 items)
- Lasso-10 [223] (30 items)
- Measure for Intention to Adhere to HIV Treatment [224]* (14 items)
- Medication Adherence Self-Reports in Adults with Type 2 Diabetes [225]* (6 items)
- Medication Adherence Survey for Hemodialysis Patients [226]* (23 items)
- Multiple Sclerosis Treatment Adherence Questionnaire (MS-TAQ) [227] (11 items)
- Outcome Expectations for Osteoporosis Medication Adherence Scale in Chinese Immigrants (OEOMA-C) [228]** (5 items)
- Patient-Reported Measures Assessing Adherence Behaviors and Barriers in Patients Living with HIV [229]* (7 items)
- Perceived Barriers to Antiretroviral Therapy Adherence (PEDIA) Scale [230]** (18 items)
- Portuguese End-Stage Renal Disease Questionnaire (PESRD-AQ) [231]*** (46 items)
- Questionnaire for Adherence with Topical Treatments in Psoriasis (QATOP) [232] (9 items)
- Reduced Glaucoma Treatment Compliance Assessment Tool (GTCAT) [233,234] (28 items)
- Risk of Nonadherence to Antibiotic Treatment Questionnaire [235]* (20 items)
- Self-Assessment Tool to Measure Imatinib Adherence in Patients with Chronic Myeloid Leukemia [236]* (10 items)
- Self-Efficacy for Osteoporosis Medication Adherence Scale in Chinese Immigrants (SEOMA-C) [228]** (14 items)
- Self-Report Measures in Assessing Antiretroviral Adherence [237]* (3 items)
- Self-Reported Compliance to Metered-Dose Inhalers Questionnaire [238]* (4 items)
- Self-Reported Questionnaire Assessing Adherence to Antiretroviral Medication [239]* (5 items)
- Test of the Adherence to Inhalers (TAI) [240] (12 items)
- Treatment Adherence Survey – Patient Version (TAS-P) [241] (16 items)

### Item Evaluation

At the stage of winnowing, 319 items inconsistent with the definitions of the domains or subdomains were removed. The 2 independent reviewers achieved a 90.4% agreement on the items for removal. Most eliminated items were found to be highly disease-specific (eg,“I arrange my oral antidiabetic medication or insulin dose myself according to my food intake.”) or open-ended (eg,“How many doses did you miss?”).

In the Extent of Adherence, a total of 77 representative items were identified, while 344 items were included in the Reasons for Nonadherence, with an average of 70 items (SD 43.1; range 24-137) within each of the 5 dimensions. The breakdown of the number of items in each domain and subdomain is summarised in Table 2, and a representative table of the mapping of each item is presented in Multimedia Appendix 3. Binned and winnowed items that were granted approval by the study investigators or PROM developers to be openly listed in the item bank are presented in Multimedia Appendix 4.

**Table 2:** Summary of domains and the number of items in each bin.

Domain/subdomain		Number of items	
Extent of adherence		77	
**Reasons for adherence**			
	Social and economic factors		32
	Health care team and system-related factors		24
	Condition-related factors		37
	Therapy-related factors		114
	Patient-related factors		137

## Discussion

This systematic review summarises the process of developing an item bank for the domain of general medication adherence. A multistep approach adapted from the PROMIS standards for the development of item banks [14] was used. In order to enhance the comprehensiveness of the item bank, items originating from both disease-specific and nondisease specific PROMs were considered and reviewed for inclusion. To the best of our knowledge, this is the first systematic review to collate items from various medication adherence PROMs into an item bank. As numerous PROMs have been developed and validated for use in the measurement of medication adherence, an item bank consisting of the most representative items may improve the relevance and precision of assessments [242].

The current item bank enables researchers to select items for the creation of new PROMs. The rare and highly disease-specific items were eliminated during the winnowing process; however, the items included in the item bank may be adapted for use in both specific patient groups and the general population. There have been conflicting results on the association between age and medical adherence, with some studies providing evidence of an association between younger age and nonadherence [243] and others showing an association between older age and nonadherence [244]. A CAT that was developed using IRT based on our item bank can potentially examine the changing pattern of medical adherence during a lifetime more precisely than traditional PROMs that are developed using classical test theory [245,246]. In addition, previous studies have shown that adherence to different medications may vary within the same individual [247,248]. We can potentially overcome this issue by specifying the type of medication in the question stem in the CAT.

Towards the use of the item bank in CATs, the next steps are to revise and review the items through cognitive interviews, to calibrate the items using IRT, and to evaluate the validity of test scores when the items are administered adaptively. The items that underwent binning and winnowing originated from various PROMs. They were created in varying styles, phrasings, and sentence structures, with different response options for each item, including dichotomous, Likert, and semantic differential scales. Due to the discordances among the items, the item revision process would be prudent to facilitate the administration of the items as 1 coherent test. Once fully developed, the resulting instrument may potentially improve measurement precision and allow for a reduction in assessment time [249] and, accordingly, patient cognitive burden. Furthermore, since the scores are directly comparable [250], such an instrument would allow health care providers to measure and compare the medication adherence of a patient from consult to consult. Alongside other clinical measures, such as symptom scores and quality of life scales, these measurements may enable physicians to detect issues in a patient's medication adherence, implement timely interventions, evaluate the effectiveness of the interventions, and make prompt modifications when necessary.

This study has several strengths; 5 databases, as well as sensitive search filters, were used to capture as many potentially relevant articles as possible. The rigor of this study was established using the PRISMA statement and PROMIS standards. The PRISMA statement was used because it enhances the transparency and clarity of systematic reviews [251]. The utilization of modern statistical methods to improve the functionality of PROMs has elevated the expectations of instruments beyond robust psychometric properties. As such, the PROMIS standards were adapted for use as they endorse the minimum standards for PROMs used in patient-centered health outcomes and facilitate the development of common metrics for accurate comparisons across conditions, healthcare systems, and geographical locations [252].

This systematic review has some limitations. Firstly, we only included full-text articles in English. Full-text articles were necessary as they are peer-reviewed and recommended by Terwee et al [253]. Nonetheless, the 12 foreign-language articles that would otherwise be eligible for full-text review made up only 3.9% of the included articles. Secondly, the item bank does not include items developed by the 160 authors (75.1% of the articles assessed for inclusion) who either chose not to consent to the inclusion of their PROMs or were uncontactable. Of note, PROMs included in the item bank have adequately captured the concepts measured by the majority of items for which consent was not obtained. To ensure that no range of concepts has been overlooked from the exclusion of the studies, future studies can consider conducting interviews with patients, as well as conducting expert opinion reviews to elicit the conceptual model for medical adherence relevant to the local context. Thirdly, this systematic review only included validation studies of medication adherence PROMs. This was deemed appropriate given the importance of validation in justifying the use of the instrument [254]. Well-validated adherence scales have been strongly correlated with objective measures of adherence [255], allowing accurate and reliable assessment of medication adherence. In addition, expert review and patient feedback were not sought as part of the item evaluation process in this study. As the concept of medical adherence may differ between different sociocultural contexts [256,257], different countries should perform an expert review and gather patient feedback through cognitive interviews based on this item bank to ensure the development of a culturally sensitive instrument to measure medication adherence.

In conclusion, this study has identified and collated the items from 47 unique medication adherence PROMs into an item bank through a systematic review. Researchers are able to select appropriate items from the item bank for the creation of new PROMs. Future research may consider revising and reviewing the items through cognitive interviews, calibrating the items through IRT, and developing a CAT to measure medication adherence precisely.

## Appendix

Multimedia Appendix 1 Search strategy.

Multimedia Appendix 2 Studies excluded from the item bank due to a lack of consent.

Multimedia Appendix 3 Item mapping of domains and subdomains.

Multimedia Appendix 4 Item bank to measure the extent of and reasons for medication adherence.

## Abbreviations

CAT: computer-adaptive tests
DOSE-Nonadherence: Domains of Subjective Extent of Nonadherence
IRT: item response theory
PROMs: patient-reported outcome measures
PRISMA: Preferred reporting items for systematic review and meta-analysis
PROMIS: Patient-Reported Outcomes Measurement Information System
