# Supplementary material for: Development of an Item Bank to Measure Medication Adherence: Systematic Review
Source: J Med Internet Res. 2020 Oct 8;22(10):e19089. doi: 10.2196/19089 (PMC7582150; doi:10.2196/19089)
Supplement: Multimedia Appendix 2 [file jmir_v22i10e19089_app2.docx]

| Author(s) | Title of Study |
| --- | --- |
| A. A. Irvine, J. T. Saunders, M. B. Blank and W. R. Carter | Validation of scale measuring environmental barriers to diabetes-regimen adherence [33] |
| A. B. Lewin, A. M. LaGreca, G. R. Geffken, L. B. Williams, D. C. Duke, E. A. Storch and J. H. Silverstein | Validity and reliability of an adolescent and parent rating scale of type 1 diabetes adherence behaviors: the Self-Care Inventory (SCI) [34] |
| A. C. Moss, Y. Lillis, J. B. Edwards George, N. K. Choudhry, A. H. Berg, A. S. Cheifetz, G. Horowitz and D. A. Leffler | Attitudes to mesalamine questionnaire: A novel tool to predict mesalamine nonadherence in patients with IBD [35] |
| A. D. de Oliveira, D. E. Morisky, S. J. F. Neves, F. A. Costa and D. P. de Lyra | The 8-item Morisky Medication Adherence Scale: Validation of a Brazilian-Portuguese version in hypertensive adults [36] |
| A. Jerant, R. Dimatteo, J. Arnsten, M. Moore-Hill and P. Franks | Self-report adherence measures in chronic illness: Retest reliability and predictive validity [37] |
| A. L. Dima, A. M. Schweitzer, R. Diaconiţ, E. Remor and R. S. Wanless | Adherence to ARV medication in Romanian young adults: self-reported behaviour and psychological barriers [38] |
| A. L. Dima, E. van Ganse, L. Laforest, N. Texier and M. de Bruin | Measuring medication adherence in asthma: Development of a novel self-report tool [39] |
| A. Najimi, F. Mostafavi, G. Sharifirad and P. Golshiri | Development and study of self-efficacy scale in medication adherence among Iranian patients with hypertension [40] |
| A. Regnault, M. Viala-Danten, H. Gilet and G. Berdeaux | Scoring and psychometric properties of the Eye-Drop Satisfaction Questionnaire (EDSQ), an instrument to assess satisfaction and compliance with glaucoma treatment [41] |
| A. S. Athavale, J. P. Bentley, B. F. Banahan, 3rd, D. J. McCaffrey, 3rd and P. F. Pace | Preliminary development of the Medication Nonpersistence Scale [42] |
| A. Turcu-știolică, M. Bogdan, A. E. Tăerel, A. Boboia, M. S. Subțirelu, L. G. Foia and V. Pădureanu | Developing of a new tool for evaluation of therapeutic adherence in COPD [43] |
| A. Zeller, K. Schroeder and T. J. Peters | An adherence self-report questionnaire facilitated the differentiation between nonadherence and nonresponse to antihypertensive treatment [44] |
| A. Zongo, L. Guénette, J. Moisan and J. P. Grégoire | Predictive Validity of Self-Reported Measures of Adherence to Noninsulin Antidiabetes Medication against Control of Glycated Hemoglobin Levels [45] |
| A. Zongo, L. Guenette, J. Moisan, L. Guillaumie, S. Lauzier and J. P. Gregoire | Revisiting the internal consistency and factorial validity of the 8-item Morisky Medication Adherence Scale [46] |
| A. Zweben, M. E. Piepmeier, L. Fucito and S. S. O'Malley | The clinical utility of the Medication Adherence Questionnaire (MAQ) in an alcohol pharmacotherapy trial [47] |
| B. J. Feldman, R. J. Fredericksen, P. K. Crane, S. A. Safren, M. J. Mugavero, J. H. Willig, J. M. Simoni, I. B. Wilson, M. S. Saag, M. M. Kitahata and H. M. Crane | Evaluation of the single-item self-rating adherence scale for use in routine clinical care of people living with HIV [48] |
| B. Jankowska-Polanska, I. Uchmanowicz, A. Chudiak, K. Dudek, D. E. Morisky and A. Szymanska-Chabowska | Psychometric properties of the Polish version of the eight-item Morisky Medication Adherence Scale in hypertensive adults [49] |
| B. L. Svarstad, B. A. Chewning, B. L. Sleath and C. Claesson | The Brief Medication Questionnaire: a tool for screening patient adherence and barriers to adherence [50] |
| B. Resnick, L. Wehren and D. Orwig | Reliability and validity of the self-efficacy and outcome expectations for osteoporosis medication adherence scales [51] |
| B. Spire, B. Arnould, F. Barbier, J. Durant, J. Gilquin, R. Landman, S. Carret, C. Saussier, S. El Kebir and I. Cohen-Codar | Simplification and first validation of a short battery of patient questionnaires for clinical management of HIV-infected patients: The HIV-SQUAD (symptom quality of life adherence) questionnaire® [52] |
| C. De Las Cuevas and W. Penate | Psychometric properties of the eight-item Morisky Medication Adherence Scale (MMAS-8) in a psychiatric outpatient setting [53] |
| C. M. Brooks, J. M. Richards, C. L. Kohler, S. J. Soong, B. Martin, R. A. Windsor and W. C. Bailey | Assessing adherence to asthma medication and inhaler regimens: a psychometric analysis of adult self-report scales [54] |
| C. Mahler, K. Hermann, R. Horne, S. Ludt, W. E. Haefeli, J. Szecsenyi and S. Jank | Assessing reported adherence to pharmacological treatment recommendations. Translation and evaluation of the Medication Adherence Report Scale (MARS) in Germany [8] |
| C. Tan, G. G. Teng, K. J. Chong, P. P. Cheung, A. Lim, H. L. Wee and A. Santosa | Utility of the Morisky Medication Adherence Scale in gout: a prospective study [55] |
| C. Willey, C. Redding, J. Stafford, F. Garfield, S. Geletko, T. Flanigan, K. Melbourne, J. Mitty and J. J. Caro | Stages of change for adherence with medication regimens for chronic disease: development and validation of a measure [56] |
| C. Y. Lin, H. T. Ou, M. Nikoobakht, A. Brostrom, K. Arestedt and A. H. Pakpour | Validation of the 5-Item Medication Adherence Report Scale in Older Stroke Patients in Iran [57] |
| C. Y. Osborn and J. S. Gonzalez | Measuring insulin adherence among adults with type 2 diabetes [58] |
| D. E. Morisky, A. Ang, M. Krousel-Wood and H. J. Ward | Predictive validity of a medication adherence measure in an outpatient setting [59] |
| D. E. Morisky, L. W. Green and D. M. Levine | Concurrent and predictive validity of a self-reported measure of medication adherence [60] |
| D. Rofail, L. Abetz, M. Viala, C. Gait, J. F. Baladi and K. Payne | Satisfaction and adherence in patients with iron overload receiving iron chelation therapy as assessed by a newly developed patient instrument [61] |
| D. S. Shin and C. J. Kim | Psychometric evaluation of a Korean version of the 8-item Medication Adherence Scale in rural older adults with hypertension [62] |
| De Geest, S., Abraham, L., Gemoets, H., & Evers, G. | Development of the long-term medication behaviour self efficacy scale: qualitative study for item development [63] |
| E. de Klerk, D. van der Heijde, H. van der Tempel and S. van der Linden | Development of a questionnaire to investigate patient compliance with antirheumatic drug therapy [64] |
| E. J. Blumberg, M. F. Hovell, N. J. Kelley, A. Y. Vera, C. L. Sipan and J. P. Berg | Self-report INH adherence measures were reliable and valid in Latino adolescents with latent tuberculosis infection [65] |
| E. J. Unni and K. B. Farris | Development of a new scale to measure self-reported medication nonadherence [66] |
| E. J. Unni, J. L. Olson and K. B. Farris | Revision and validation of Medication Adherence Reasons Scale (MAR-Scale) [67] |
| E. J. Unni, N. Sternbach and A. Goren | Using the Medication Adherence Reasons Scale (MAR-Scale) to identify the reasons for non-adherence across multiple disease conditions [68] |
| E. Salt, L. Hall, A. R. Peden and R. Home | Psychometric properties of three medication adherence scales in patients with rheumatoid arthritis [69] |
| E. Tommelein, E. Mehuys, I. Van Tongelen, G. Brusselle and K. Boussery | Accuracy of the Medication Adherence Report Scale (MARS-5) as a quantitative measure of adherence to inhalation medication in patients with COPD [70] |
| F. Alhomoud, F. Alhomoud and I. Millar | How effectively are your patients taking their medicines? A critical review of the Strathclyde Compliance Risk Assessment Tool in relation to the 'MMAS' and 'MARS' [71] |
| F. C. Vale, E. T. Santa-Helena, M. A. Santos, Wmdes Carvalho, P. R. Menezes, C. R. Basso, M. H. Silva, A. M. Alves and M. I. B. Nemes | Development and validation of the WebAd-Q Questionnaire to monitor adherence to HIV therapy [72] |
| F. I. Cinar, M. Cinar, S. Yilmaz, C. Acikel, H. Erdem, S. Pay and I. Simsek | Cross-cultural adaptation, reliability, and validity of the turkish version of the compliance questionnaire on rheumatology (CQR-T) in patients with Behc¸et's disease [73] |
| F. J. Alsolami, X. Y. Hou, I. Correa-Velez and S. M. Bahlas | An Arabic instrument to Measure Medication Adherence in Saudi Hypertensive Patients [74] |
| F. J. Ortega Suarez, J. Sanchez Plumed, M. A. Perez Valentin, P. Pereira Palomo, M. A. Munoz Cepeda and D. Lorenzo Aguiar | Validation on the simplified medication adherence questionnaire (SMAQ) in renal transplant patients on tacrolimus [75] |
| F. S. Julian, P. Martin and S. R. Erickson | Validation of the Special Projects of National Significance adherence tool in HIV/AIDS patients [76] |
| G. Fabbrini, G. Abbruzzese, P. Barone, A. Antonini, M. Tinazzi, G. Castegnaro, S. Rizzoli, D. E. Morisky, P. Lessi and R. Ceravolo | Adherence to anti-Parkinson drug therapy in the "REASON" sample of Italian patients with Parkinson's disease: the linguistic validation of the Italian version of the "Morisky Medical Adherence Scale-8 items" [77] |
| G. Fond, L. Boyer, M. Boucekine, L. A. Aden, F. Schurhoff, A. Tessier, M. Andrianarisoa, F. Berna, L. Brunel, D. Capdevielle, I. Chereau, J. Mallet, H. Denizot, J. M. Dorey, C. Dubertret, J. Dubreucq, C. Faget, F. Gabayet, R. Rey, R. Richieri, C. Passerieux, A. Schandrin, M. Urbach, P. Vidailhet, P. M. Llorca and D. Misdrahi | Validation study of the Medication Adherence Rating Scale. Results from the FACE-SZ national dataset [78] |
| G. O. Owie, S. O. Olotu and B. O. James | Reliability and validity of the Medication Adherence Rating Scale in a cohort of patients with schizophrenia from Nigeria [79] |
| G. Ogedegbe, C. A. Mancuso, J. P. Allegrante and M. E. Charlson | Development and evaluation of a medication adherence self-efficacy scale in hypertensive African-American patients [80] |
| H. Cate, D. Bhattacharya, A. Clark, R. Holland and D. C. Broadway | A comparison of measures used to describe adherence to glaucoma medication in a randomised controlled trial [81] |
| H. Jonsdottir, S. Opjordsmoen, A. B. Birkenaes, J. A. Engh, P. A. Ringen, A. Vaskinn, T. O. Aamo, S. Friis and O. A. Andreassen | Medication adherence in outpatients with severe mental disorders: relation between self-reports and serum level [82] |
| H. W. Cohen, C. Shmukler, R. Ullman, C. M. Rivera and E. A. Walker | Measurements of medication adherence in diabetic patients with poorly controlled HbA(1c) [83] |
| I. Arnet, C. Metaxas, P. N. Walter, D. E. Morisky and K. E. Hersberger | The 8-item Morisky Medication Adherence Scale translated in German and validated against objective and subjective polypharmacy adherence measures in cardiovascular patients [84] |
| I. Sadakathulla, U. V. Mateti, A. Kellarai and K. Bhat | Adhering to antihypertensive treatment is vitally important [85] |
| I. Zschocke, U. Mrowietz, A. Lotzin, E. Karakasili and K. Reich | Assessing adherence factors in patients under topical treatment: development of the Topical Therapy Adherence Questionnaire (TTAQ) [86] |
| Iv S. B. Kennedy | Developing a self-administered tool to predict adherence to antiretroviral therapy: Design, method, and objectives [87] |
| J. C. Prado, Jr., E. Kupek and D. Mion, Jr. | Validity of four indirect methods to measure adherence in primary care hypertensives [88] |
| J. George, T. Vuong, M. J. Bailey, D. C. M. Kong, J. L. Marriott and K. Stewart | Medication regimen complexity and adherence in patients at risk of medication misadventure [89] |
| J. H. Arnsten, P. A. Demas, H. Farzadegan, R. W. Grant, M. N. Gourevitch, C. J. Chang, D. Buono, H. Eckholdt, A. A. Howard and E. E. Schoenbaum | Antiretroviral therapy adherence and viral suppression in HIV-infected drug users: comparison of self-report and electronic monitoring [6] |
| J. H. Kim, W. Y. Lee, Y. P. Hong, W. S. Ryu, K. J. Lee, W. S. Lee and D. E. Morisky | Psychometric properties of a short self-reported measure of medication adherence among patients with hypertension treated in a busy clinical setting in Korea [90] |
| J. Koschack, G. Marx, J. Schnakenberg, M. M. Kochen and W. Himmel | Comparison of two self-rating instruments for medication adherence assessment in hypertension revealed insufficient psychometric properties [91] |
| J. L. Cohen, D. M. Mann, J. P. Wisnivesky, R. Home, H. Leventhal, T. J. Musumeci-Szabó and E. A. Halm | Assessing the validity of self-reported medication adherence among inner-city asthmatic adults: the Medication Adherence Report Scale for Asthma [92] |
| J. M. Simoni, D. Huh, Y. Wang, I. B. Wilson, N. R. Reynolds, R. H. Remien, K. Goggin, R. Gross, M. I. Rosen, N. Schneiderman, J. Arnsten, C. E. Golin, J. A. Erlen, D. R. Bangsberg and H. Liu | The validity of self-reported medication adherence as an outcome in clinical trials of adherence-promotion interventions: Findings from the MACH14 study [93] |
| J. P. Nordmann, P. Denis, M. Vigneux, E. Trudeau, I. Guillemin and G. Berdeaux | Development of the conceptual framework for the Eye-Drop Satisfaction Questionnaire (EDSQ) in glaucoma using a qualitative study [94] |
| J. Risser, T. A. Jacobson and S. Kripalani | Development and psychometric evaluation of the Self-efficacy for Appropriate Medication Use Scale (SEAMS) in low-literacy patients with chronic disease [95] |
| J. van den Boogaard, R. A. Lyimo, M. J. Boeree, G. S. Kibiki and R. E. Aarnoutse | Electronic monitoring of treatment adherence and validation of alternative adherence measures in tuberculosis patients: a pilot study [96] |
| J. Y. Lee, S. Y. Lee, H. J. Hahn, I. J. Son, S. G. Hahn and E. B. Lee | Cultural adaptation of a compliance questionnaire for patients with rheumatoid arthritis to a Korean version [97] |
| Jansà M, Vidal M, Giménez M, Conget I, Galindo M, Roca D | Psychometric analysis of the Spanish and Catalan versions of the Diabetes Self-Care inventory-revised version questionnaire [98] |
| K. Chaiyachati, L. R. Hirschhorn, F. Tanser, M. L. Newell and T. Bärnighausen | Validating five questions of antiretroviral nonadherence in a public-sector treatment program in rural South Africa [99] |
| K. E. Wickersham, S. M. Sereika, H. J. Kang, L. K. Tamres and J. A. Erlen | Use of a Self-Report Medication Adherence Scale for Measuring Adherence to Antiretroviral Therapy in Patients With HIV/AIDS [100] |
| K. Kosilov, S. Loparev, I. Kuzina, L. Kosilova, M. Ivanovskaya and A. Prokofyeva | Effectiveness of a new tool for self-evaluation of adherence to antimuscarinic drug treatment in older patients of both sexes with urge incontinence [101] |
| K. Kosilov, S. Loparev, I. Kuzina, O. Shakirova, N. Zhuravskaya and A. Lobodenko | Self-assessment of treatment compliance with antimuscarinic drugs and lower urinary tract condition among women with urinary incontinence [102] |
| K. Kosilov, S. Loparev, I. Kuzina, O. Shakirova, N. Zhuravskaya and A. Lobodenko | The effective tool for self-assessment of adherence to treatment in patients with benign prostatic obstruction and overactive bladder symptoms [103] |
| K. M. Walewski, L. Cicutto, A. D. D'Urzo, R. J. Heslegrave and K. R. Chapman | Evaluation of a Questionnaire to Assess Compliance with Anti-asthma Medications [104] |
| K. Reynolds, H. N. Viswanathan, C. D. O'Malley, P. Muntner, T. N. Harrison, T. C. Cheetham, J. W. Hsu, D. T. Gold, S. Silverman, A. Grauer and D. E. Morisky | Psychometric properties of the Osteoporosis-specific Morisky Medication Adherence Scale in postmenopausal women with osteoporosis newly treated with bisphosphonates [105] |
| K. Reynolds, H. N. Viswanathan, P. Muntner, T. N. Harrison, T. C. Cheetham, J. W. Hsu, D. T. Gold, S. Silverman, A. Grauer, D. E. Morisky and C. D. O'Malley | Validation of the Osteoporosis-Specific Morisky Medication Adherence Scale in long-term users of bisphosphonates [106] |
| K. Thompson, J. Kulkarni and A. A. Sergejew | Reliability and validity of a new Medication Adherence Rating Scale (MARS) for the psychoses [107] |
| K. V. Kosilov, S. A. Loparev, I. G. Kuzina, O. V. Shakirova, Y. I. Gainullina, L. V. Kosilova and A. S. Prokofyeva | A new tool for self-evaluation of adherence to antimuscarinic drugs treatment in patients with urinary incontinence [108] |
| K. Zemmour, A. Tinland, M. Boucekine, V. Girard, S. Loubiere, N. Resseguier, G. Fond, P. Auquier and L. Boyer | Validation of the Medication Adherence Rating Scale in homeless patients with schizophrenia: Results from the French Housing First experience [109] |
| Kyngäs, H. A., Skaar-Chandler, C. A., & Duffy, M. E. | The development of an instrument to measure the compliance of adolescents with a chronic disease [110] |
| L. Fialko, P. A. Garety, E. Kuipers, G. Dunn, P. E. Bebbington, D. Fowler and D. Freeman | A large-scale validation study of the Medication Adherence Rating Scale (MARS) [111] |
| L. Guénette, J. Moisan, M. Préville and R. Boyer | Measures of adherence based on self-report exhibited poor agreement with those based on pharmacy records [112] |
| L. Lee, S. El-Den, R. Horne and S. R. Carter | Patient satisfaction with information, concerns, beliefs and adherence to topical corticosteroids [113] |
| L. McDonald-Miszczak, P. Maris, T. Fitzgibbon and G. Ritchie | A pilot study examining older adults' beliefs related to medication adherence: the BERMA survey [114] |
| L. Nogueira-Silva, A. Sá-Sousa, M. J. Lima, A. Monteiro, C. Dennison-Himmelfarb and J. A. Fonseca | Translation and cultural adaptation of the Hill-Bone Compliance to High Blood Pressure Therapy Scale to Portuguese [115] |
| L. S. Matza, J. Park, K. S. Coyne, E. P. Skinner, K. G. Malley and R. Q. Wolever | Derivation and validation of the ASK-12 adherence barrier survey [116] |
| Lubinga SJ, Millar I, Babigumira JB | Pilot evaluation of the psychometric properties of a self-medication Risk Assessment Tool among elderly patients in a community setting [117] |
| M. A. Chesney, J. R. Ickovics, D. B. Chambers, A. L. Gifford, J. Neidig, B. Zwickl and A. W. Wu | Self-reported adherence to antiretroviral medications among participants in HIV clinical trials: the AACTG Adherence Instruments [118] |
| M. A. Chisholm, C. E. Lance, G. M. Williamson and L. L. Mulloy | Development and validation of an immunosuppressant therapy adherence barrier instrument [119] |
| M. A. S. Ali, D. A. E. Abou-Taleb and R. R. Mohamed | Treatment adherence and beliefs about medicines among Egyptian vitiligo patients [120] |
| M. Alsous, F. Alhalaiqa, R. Abu Farha, M. Abdel Jalil, J. McElnay and R. Horne | Reliability and validity of Arabic translation of Medication Adherence Report Scale (MARS) and Beliefs about Medication Questionnaire (BMQ)-specific for use in children and their parents [121] |
| M. Axelsson, L. Ekerljung, B. Lundback and J. Lotvall | Personality and unachieved treatment goals related to poor adherence to asthma medication in a newly developed adherence questionnaire - a population-based study [122] |
| M. Dibonaventura, N. Wintfeld, J. Huang and A. Goren | The association between nonadherence and glycated hemoglobin among type 2 diabetes patients using basal insulin analogs[123] |
| M. Fahey, A. Abdulmajeed and K. Sabra | Measurement of adherence to anti-hypertensive medication as perceived by doctors and patients [124] |
| M. J. Kikkert, M. W. Koeter, J. J. Dekker, L. Burti, D. Robson, B. Puschner and A. H. Schene | The predictive validity of subjective adherence measures in patients with schizophrenia [125] |
| M. Kleppe, J. Lacroix, J. Ham and C. Midden | The development of the ProMAS: A probabilistic medication adherence scale [126] |
| M. Krousel-Wood, C. Joyce, E. W. Holt, E. B. Levitan, A. Dornelles, L. S. Webber and P. Muntner | Development and evaluation of a self-report tool to predict low pharmacy refill adherence in elderly patients with uncontrolled hypertension [127] |
| M. Krousel-Wood, P. Muntner, A. Jannu, K. Desalvo and R. N. Re | Reliability of a medication adherence measure in an outpatient setting [128] |
| M. Lu, S. A. Safren, P. R. Skolnik, W. H. Rogers, W. Coady, H. Hardy and I. B. Wilson | Optimal recall period and response task for self-reported HIV medication adherence [129] |
| M. Nakhaeizadeh and A. Khalooei | Psychometric Properties of Persian version of the 8-item Morisky Medication Adherence Scale in Type 2 Diabetes Patients [130] |
| M. Saffari, I. M. Zeidi, B. Fridlund, H. Chen and A. H. Pakpour | A Persian Adaptation of Medication Adherence Self-Efficacy Scale (MASES) in Hypertensive Patients: Psychometric Properties and Factor Structure [131] |
| M. T. Kim, M. N. Hill, L. R. Bone and D. M. Levine | Development and testing of the Hill-Bone Compliance to High Blood Pressure Therapy Scale [132] |
| Mannheimer SB, Mukherjee R, Hirschhorn LR | The CASE adherence index: A novel method for measuring adherence to antiretroviral therapy [133] |
| N. A. Duncan, W. G. Kronenberger, C. P. Roberson and A. D. Shapiro | VERITAS-PRN: a new measure of adherence to episodic treatment regimens in haemophilia [134] |
| N. Duncan, W. Kronenberger, C. Roberson and A. Shapiro | VERITAS-Pro: a new measure of adherence to prophylactic regimens in haemophilia [135] |
| N. R. Reynolds, J. Sun, H. N. Nagaraja, A. L. Gifford, A. W. Wu and M. A. Chesney | Optimizing measurement of self-reported adherence with the ACTG Adherence Questionnaire: a cross-protocol analysis [136] |
| N. van de Steeg, M. Sielk, M. Pentzek, C. Bakx and A. Altiner | Drug-adherence questionnaires not valid for patients taking blood-pressure-lowering drugs in a primary health care setting [137] |
| O. A. Sowunmi and P. O. Onifade | Psychometric evaluation of medication adherence rating scale (MARS) among Nigerian patients with schizophrenia [138] |
| O. Kampman, K. Lehtinen and V. Lassila | The reliability of compliance assessments performed by doctors and patients during neuroleptic treatment: a comparison of compliance ratings [139] |
| O. Marsicano Ede, S. Fernandes Nda, F. Colugnati, F. R. Grincenkov, N. M. Fernandes, S. De Geest and H. Sanders-Pinheiro | Transcultural adaptation and initial validation of Brazilian-Portuguese version of the Basel assessment of adherence to immunosuppressive medications scale (BAASIS) in kidney transplants [140] |
| P. Ambarish, B. Stephanie, A. Colby, A. Debbie, P. Angela, D. Sandeep and H. Ethan | Comparison of morisky medication adherence scale with therapeutic drug monitoring in resistant hypertension [141] |
| P. Sakthong, R. Chabunthom and R. Charoenvisuthiwongs | Psychometric properties of the Thai version of the 8-item Morisky Medication Adherence Scale in patients with type 2 diabetes [142] |
| P. W. Choo, C. S. Rand, T. S. Inui, M. L. Lee, E. Cain, M. Cordeiro-Breault, C. Canning and R. Platt | Validation of patient reports, automated pharmacy records, and pill counts with electronic monitoring of adherence to antihypertensive therapy [143] |
| P. W. Garcia-Marcos, P. L. Brand, A. A. Kaptein and T. Klok | Is the MARS questionnaire a reliable measure of medication adherence in childhood asthma? [144] |
| R. Atsuta, Y. To, S. Sakamoto, I. Mukai, A. Kobayashi, A. Kinoshita and K. Takahashi | Assessing usability of the “Adherence Starts with Knowledge 20” (ASK-20) questionnaire for Japanese adults with bronchial asthma receiving inhaled corticosteroids long term [145] |
| R. Ayiesah, J. H. Leonard and C. Y. Chong | Development and validation of non-adherence to pulmonary rehabilitation questionnaire: a clinical tool for patients with chronic obstructive pulmonary diseases [146] |
| R. B. Pedrosa and R. C. Rodrigues | Adaptation and evaluation of the measurement properties of the Brazilian version of the Self-efficacy for Appropriate Medication Adherence Scale [147] |
| R. C. Rathbun, K. C. Farmer, S. M. Lockhart and J. R. Stephens | Validity of a stage of change instrument in assessing medication adherence in indigent patients with HIV infection [148] |
| R. Fernandes, S. Wales, J. Crisp and H. Kyngas | Modification and testing of the chronic disease compliance instrument to measure treatment compliance in adolescents with diabetes [149] |
| R. Jacobsen, C. Møldrup, L. Christrup, P. Sjøgren and O. B. Hansen | The Danish version of the medication adherence report scale: Preliminary validation in cancer pain patients [150] |
| R. Sampaio, L. F. Azevedo, C. C. Dias, R. Horne and J. M. Castro Lopes | Portuguese version of the Medication Adherence Report Scale (MARS-9): Validation in a population of chronic pain patients [151] |
| Rbds Pedrosa, R. C. M. Rodrigues, H. C. Oliveira and N. M. C. Alexandre | Construct Validity of the Brazilian Version of the Self-Efficacy for Appropriate Medication Adherence Scale [152] |
| S. A. Kristina, L. R. Putri, D. A. Riani, Z. Ikawati and D. Endarti | Validity of self-reported measure of medication adherence among diabetic patients in Indonesia [153] |
| S. A. R. Shilbayeh, W. A. Almutairi, S. A. Alyahya, N. H. Alshammari, E. Shaheen and A. Adam | Validation of knowledge and adherence assessment tools among patients on warfarin therapy in a Saudi hospital anticoagulant clinic [154] |
| S. C. Kalichman, C. M. Amaral, C. Swetzes, M. Jones, R. Macy, M. O. Kalichman and C. Cherry | A simple single-item rating scale to measure medication adherence: further evidence for convergent validity [155] |
| S. C. Kalichman, D. Cain, A. Fuhrel, L. Eaton, K. Di Fonzo and T. Ertl | Assessing medication adherence self-efficacy among low-literacy patients: development of a pictographic visual analogue scale [156] |
| S. E. Wilks, C. A. Spivey and M. A. Chisholm-Burns | Psychometric re-evaluation of the immunosuppressant therapy adherence scale among solid-organ transplant recipients [157] |
| S. Fernandez, W. Chaplin, A. M. Schoenthaler and G. Ogedegbe | Revision and validation of the medication adherence self-efficacy scale (MASES) in hypertensive African Americans [158] |
| S. J. Bennett, L. B. Milgrom, V. Champion and G. A. Huster | Beliefs about medication and dietary compliance in people with heart failure: an instrument development study [159] |
| S. J. Shalansky, A. R. Levy and A. P. Ignaszewski | Self-reported Morisky score for identifying nonadherence with cardiovascular medications [160] |
| S. Koneru, M. Shishov, A. Ware, Y. Farhey, A. B. Mongey, T. B. Graham, M. H. Passo, J. L. Houk, G. C. Higgins and H. I. Brunner | Effectively measuring adherence to medications for systemic lupus erythematosus in a clinical setting [161] |
| S. Matza Louis, S. Yu-Isenberg Kristina, S. Coyne Karin, Park Jinhee, Wakefield Jessica, P. Skinner Elizabeth, Wolever Ruth Quillian, Louis S. Matza, Kristina S. Yu-Isenberg, Karin S. Coyne, Jinhee Park, Jessica Wakefield, Elizabeth P. Skinner and Ruth QuillianWolever | Further testing of the reliability and validity of the ASK-20 adherence barrier questionnaire in a medical center outpatient population [162] |
| S. Mueller, T. Wilke, V. Gorasso, M. Erhart and J. M. Kittner | Adaption and validation of the adherence barriers questionnaire for HIV patients on antiretroviral therapy (ABQ-HIV) [163] |
| S. Müller, T. Kohlmann and T. Wilke | Validation of the Adherence Barriers Questionnaire - an instrument for identifying potential risk factors associated with medication-related non-adherence [164] |
| S. Okello, B. Nasasira, A. N. Muiru and A. Muyingo | Validity and Reliability of a Self-Reported Measure of Antihypertensive Medication Adherence in Uganda [165] |
| S. R. Hahn, J. Park, E. P. Skinner, K. S. Yu-Isenberg, M. B. Weaver, B. Crawford and P. W. Flowers | Development of the ASK-20 adherence barrier survey [166] |
| S. R. Smith, A. S. Wahed, S. S. Kelley, H. S. Conjeevaram, P. R. Robuck and M. W. Fried | Assessing the validity of self-reported medication adherence in hepatitis C treatment [167] |
| S. Sidorkiewicz, V. T. Tran, C. Cousyn, E. Perrodeau and P. Ravaud | Development and validation of an instrument to assess treatment adherence for each individual drug taken by a patient [168] |
| S. Sriwarakorn, S. Krittiyanunt and R. Sakulbumrungsil | SENSITIVITY AND SPECIFICITY OF THAI-VERSION BRIEF MEDICATION QUESTIONNAIRE [169] |
| S. Stjernsward, K. Persson, R. Nielsen, E. Tuninger and S. Levander | A modified Drug Attitude Inventory used in long-term patients in sheltered housing [170] |
| S. T. Ashur, K. Shamsuddin, S. A. Shah, S. Bosseri and D. E. Morisky | Reliability and known-group validity of the Arabic version of the 8-item Morisky Medication Adherence Scale among type 2 diabetes mellitus patients [171] |
| S. Wales, J. Crisp, R. Fernandes and H. Kyngas | Modification and testing of the chronic disease compliance instrument to measure treatment compliance in adolescents with asthma [172] |
| Song, H.-J., Song, Y., Han, H.-R., Nam, S., Nguyen, T., & Kim, M. T. | Psychometric Evaluation of Hill-Bone Medication Adherence Subscale [10] |
| T. Kerr, R. S. Hogg, B. Yip, M. W. Tyndall, J. Montaner and E. Wood | Validity of self-reported adherence among injection drug users [173] |
| T. Mumtaz, S. A. Haider, J. A. Malik and A. M. La Greca | Translation, validation and effectiveness of self-care inventory in assessing adherence to diabetes treatment [174] |
| T. P. Giordano, D. Guzman, R. Clark, E. D. Charlebois and D. R. Bangsberg | Measuring adherence to antiretroviral therapy in a diverse population using a visual analogue scale [175] |
| T. P. Hogan, A. G. Awad and R. Eastwood | A self-report scale predictive of drug compliance in schizophrenics: reliability and discriminative validity [176] |
| T. Phillips, K. Brittain, C. A. Mellins, A. Zerbe, R. H. Remien, E. J. Abrams, L. Myer and I. B. Wilson | A Self-Reported Adherence Measure to Screen for Elevated HIV Viral Load in Pregnant and Postpartum Women on Antiretroviral Therapy [177] |
| U. U. Andy, H. S. Harvie, A. L. Smith, K. J. Propert, H. R. Bogner and L. A. Arya | Validation of a self-administered instrument to measure adherence to anticholinergic drugs in women with overactive bladder [178] |
| V. Breuil, B. Cortet, F. E. Cotte, B. Arnould, C. Dias-Barbosa, A. F. Gaudin, A. Regnault, A. R. de Climens and E. Legrand | Validation of the adherence evaluation of osteoporosis treatment (ADEOS) questionnaire for osteoporotic post-menopausal women [179] |
| V. Korb-Savoldelli, F. Gillaizeau, J. Pouchot, E. Lenain, N. Postel-Vinay, P. F. Plouin, P. Durieux and B. Sabatier | Validation of a French version of the 8-item Morisky medication adherence scale in hypertensive adults [180] |
| Vreeman RC, Nyandiko WM, Ayaya SO, Walumbe EG, Inui TS. | Cognitive interviewing for cross-cultural adaptation of pediatric antiretroviral therapy adherence measurement items [181] |
| W. Da, X. Li, S. Qiao, Y. Zhou and Z. Shen | Evaluation of self-report adherence measures and their associations with detectable viral load among people living with HIV (PLHIV) in China [182] |
| W. R. Doucette, K. B. Farris, K. M. Youland, B. A. Newland, S. J. Egerton and J. M. Barnes | Development of the Drug Adherence Work-up (DRAW) tool [183] |
| W. Y. Lee, J. Ahn, J. H. Kim, Y. P. Hong, S. K. Hong, Y. T. Kim, S. H. Lee and D. E. Morisky | Reliability and validity of a self-reported measure of medication adherence in patients with type 2 diabetes mellitus in Korea [184] |
| Wei He, Ann Bonner and Debra Anderson | Patient reported adherence to hypertension treatment: A revalidation study [185] |
| Y. C. Kao and Y. P. Liu | Compliance and schizophrenia: The predictive potential of insight into illness, symptoms, and side effects [186] |
| Y. Kim, L. S. Evangelista, L. R. Phillips, C. Pavlish and J. D. Kopple | The End-Stage Renal Disease Adherence Questionnaire (ESRD-AQ): Testing The Psychometric Properties in Patients Receiving In-CenterHemodialysis[187] |
| Y. Wang, J. Lee, MphsToh, W. E. Tang and Y. Ko | Validity and reliability of a self-reported measure of medication adherence in patients with Type 2 diabetes mellitus in Singapore [188] |
| Y. Wang, M. C. Kong and Y. Ko | Psychometric properties of the 8-item Morisky Medication Adherence Scale in patients taking warfarin [189] |
