# Supplementary material for: Development of an Item Bank to Measure Medication Adherence: Systematic Review
Source: J Med Internet Res. 2020 Oct 8;22(10):e19089. doi: 10.2196/19089 (PMC7582150; doi:10.2196/19089)
Supplement: Multimedia Appendix 3 [file jmir_v22i10e19089_app3.docx]

| Items | Extent of Adherence | Reasons for Adherence | | | | |
| --- | --- | --- | --- | --- | --- | --- |
|  |  | **Social and Economic Factors** | **Healthcare Team and System-related Factors** | **Condition-related Factors** | **Therapy-related Factors** | **Patient-related Factors** |
| Do you remember to take your medication(s)? | ✓ |  |  |  | ✓ | ✓ |
| Do you change the way you take any of your medication(s) from what the doctor/nurse/pharmacist recommended? | ✓ |  |  |  |  |  |
| If you change the way you take your medication(s) what do you do differently? Please select all that apply to you:  I change how many times a day I take them. ˆ I skip some of the doses. ˆ I stop taking the medication(s). ˆ I do not take the medication(s) with/without food like the doctor/nurse/pharmacist suggested because they are too hard to take with/without food. ˆ I find some of the medication(s) too hard to take at the same time as others like the doctor/nurse/pharmacist suggested. ˆ I change some of the medication(s) that were prescribed to something I feel works the same. ˆ I do not change the way I take my medication(s). | ✓ |  |  | ✓ | ✓ | ✓ |
| I follow the doctor's instruction to take medication. | ✓ |  |  |  | ✓ | ✓ |
| I forget to take my medication. | ✓ |  |  |  | ✓ | ✓ |
| I adjust the medication and dosage based on my condition. | ✓ |  |  | ✓ | ✓ | ✓ |
| If I have the side effect of the medication, I will stop taking it. | ✓ |  |  |  | ✓ | ✓ |
| I take medication only when I am sick. | ✓ |  |  | ✓ | ✓ | ✓ |
| Over the past 7 days, I missed my medicine. | ✓ |  |  |  |  |  |
| Over the past 7 days, I skipped a dose of my medicine. | ✓ |  |  |  |  |  |
| Over the past 7 days, I did not take a dose of my medicine. | ✓ |  |  |  |  |  |
| This morning did you forget to take your medicine? | ✓ |  |  |  |  |  |
| Do you ever take your medicine too late in comparison with usual time? | ✓ |  |  |  |  |  |
| Sometimes if you feel worse when you take your medicine, do you stop taking it? | ✓ |  |  | ✓ | ✓ | ✓ |
| Do you ever not take your medicine because you forgot to do so? | ✓ |  |  |  | ✓ | ✓ |
| Do you ever miss doses of your medicine when you feel sick? | ✓ |  |  | ✓ | ✓ | ✓ |
| Does a change in your daily routine modify the way you take your medicine? | ✓ |  |  |  | ✓ | ✓ |
| Do you sometimes skip doses of your medicine when you feel better? | ✓ |  |  | ✓ | ✓ | ✓ |
| I take my pills at the same time each day. | ✓ |  |  |  |  |  |
| In the past 30 days, I was late by one hour or more in taking my medication. | ✓ |  |  |  |  |  |
| In the past 30 days, I skipped taking some or all of my medications ON PURPOSE. | ✓ |  |  |  | ✓ | ✓ |
| How much of each medicine have you missed taking in the last 7 days? | ✓ |  |  |  |  |  |
| How much extra of each medicine did you take in the last 7 days? | ✓ |  |  |  |  |  |
| Do you ever forget to take your medicine? | ✓ |  |  |  |  |  |
| Are you careless at times about taking your medicine? | ✓ |  |  |  | ✓ |  |
| Thinking about the last week. How often have you not taken your medicine? | ✓ |  |  |  |  |  |
| Over the past 3 months, how many days have you not taken any medicine at all? | ✓ |  |  |  |  |  |
| How often do you skip a dose of your medicine before you go to the doctor? | ✓ |  |  |  |  |  |
| How often do you forget to get prescriptions filled? | ✓ | ✓ | ✓ |  | ✓ | ✓ |
| How often do you forget to take your medicine? | ✓ |  |  | ✓ | ✓ | ✓ |
| How often do you decide not to take your medicine? | ✓ |  |  |  | ✓ | ✓ |
| How often do you miss taking your medicine when you feel better? | ✓ |  |  |  | ✓ | ✓ |
| How often do you miss taking your medicine when you feel sick? | ✓ |  |  | ✓ | ✓ | ✓ |
| How often do you plan ahead and refill your medicines before they run out? | ✓ | ✓ | ✓ | ✓ | ✓ | ✓ |
| In the last month, how often did you forget to take your medication(s)? | ✓ |  |  |  |  |  |
| In the last month, how often did you intend to take your medication(s) in doses different to what has been prescribed? | ✓ |  |  |  | ✓ | ✓ |
| In the last month, how often did you intend to take your medication(s) in a time different to what has been prescribed? | ✓ |  |  |  | ✓ | ✓ |
| In the last month, did you take your medication(s) with you when you are away from home (e.g., traveling or visiting relatives)? | ✓ |  |  |  | ✓ | ✓ |
| In the last month, did you stop taking your medication(s) without consulting a physician because of medication side effects? | ✓ |  |  |  |  |  |
| In the last month, did you take less of your medication (s) without consulting a physician because you feel better? | ✓ |  |  | ✓ | ✓ | ✓ |
| During sick days (e.g., flu, and diarrhea), did you take less of your medication (s) without consulting a physician due to reduced appetite? | ✓ |  |  | ✓ | ✓ | ✓ |
| In the last month, did you take less of your medication (s) without consulting a physician because of a high medication cost? | ✓ |  |  |  |  |  |
| Do you stop taking medications without informing the doctor? | ✓ |  |  |  | ✓ | ✓ |
| Do you alter medication regimen, dose and frequency by yourself? | ✓ |  |  |  | ✓ | ✓ |
| Over the past 3 weeks, I have taken the prescribed daily dosage of my medication. | ✓ |  |  |  |  |  |
| I accept the necessity of taking medication in the prescribed manner to treat my illness. | ✓ |  |  |  | ✓ | ✓ |
| I have stopped taking medication based on my own judgment (not including times when I forgot to take my medication). | ✓ |  |  | ✓ |  |  |
| During the last four weeks, how many times did you forget to take your medication? | ✓ |  |  |  |  |  |
| During the last four weeks, how many times, when you felt better, did you stop taking your medication? | ✓ |  |  | ✓ | ✓ | ✓ |
| During the last four weeks, how many times, when you felt worse, did stop taking your medication? | ✓ |  |  | ✓ | ✓ | ✓ |
| Do you ever have problems keeping time with the medicines? When? | ✓ |  |  |  |  |  |
| Some families tell us that their child worries them or makes it difficult to give them the medicines. Have you not taken medicines for any of these reasons: □ I do not know why taking am taking the medicines or keeps asking questions about the medicines □ I forgot to take medicine □ I felt ill or was vomiting  □ I was playing or at school or work  □ I refused to take medicine  □ I have problems with 1 formulation (tablets, liquids)  □ I find medicines too bitter □ I can’t take without food □ None of the above □ Other (specify) | ✓ |  |  | ✓ | ✓ | ✓ |
| In the past week,  a. On how many days did you miss at least one dose? b. On how many days did you take a dose more than an hour late?  c. How many extra doses or syringes of medicine did you take? | ✓ |  |  |  |  |  |
| How many doses of medicine did you miss in the last month? | ✓ |  |  |  |  |  |
| I skipped a dose of my medication because I was worried about its side effects. | ✓ |  |  |  | ✓ | ✓ |
| I skipped a dose of my medication because I was having side effects. | ✓ |  |  |  |  |  |
| I took a smaller amount of my medication because I was worried about its side effects. | ✓ |  |  |  |  | ✓ |
| I took a smaller amount of my medication because I was having side effects. | ✓ |  |  |  |  |  |
| I skipped a dose of my medication because I was worried about getting addicted to it. | ✓ |  |  |  | ✓ | ✓ |
| I took a smaller amount of my medication because I was worried about getting addicted to it. | ✓ |  |  |  | ✓ | ✓ |
| I skipped a dose of my medication because I was worried about costs. | ✓ | ✓ |  |  |  |  |
| I took a smaller amount of my medication because I was worried about costs. | ✓ | ✓ |  |  |  |  |
| I skipped a dose of my medication because I was feeling better. | ✓ |  |  | ✓ |  |  |
| I skipped a dose of my medication because I did not need it. | ✓ |  |  | ✓ | ✓ | ✓ |
| I took a smaller amount of my medication because I was feeling better. | ✓ |  |  | ✓ |  |  |
| I took a smaller amount of my medication because I did not need it. | ✓ |  |  | ✓ | ✓ |  |
| I forgot to take a dose of my medication. | ✓ |  |  |  |  |  |
| I missed a dose of my medication by mistake. | ✓ |  |  |  |  |  |
| I missed a dose of my medication because I did not get it refilled before I ran out. | ✓ | ✓ |  |  |  |  |
| I missed a dose of my medication because I forgot to take it with me. | ✓ |  |  |  |  |  |
| Number of daily doses and number of doses missed over the last 28 days. | ✓ |  |  |  |  |  |
| Do you just forget to take medicines some of the time? | ✓ |  |  |  | ✓ | ✓ |
| Have you taken a medicine more or less often than prescribed? | ✓ |  |  | ✓ | ✓ | ✓ |
| Have you skipped or stopped taking a medicine because you did not think it was working? | ✓ |  |  |  | ✓ | ✓ |
| Have you not had medicine with you when it was time to take it? | ✓ |  |  |  |  |  |
| Are you careless at times about taking your medicine? | ✓ |  |  |  |  | ✓ |
| Did you ever take the medication less frequently or at a smaller dose than was prescribed, or stopped the medication on your own? | ✓ |  |  |  |  |  |
| Does taking your medication(s) affect the way you live your life? |  | ✓ |  |  | ✓ | ✓ |
| Does someone (spouse/family/friends) help you remember to take your medication(s)? |  | ✓ | ✓ |  | ✓ | ✓ |
| Does your pharmacist help you with remembering to take your medication(s)? (example: dosette packs, blister packs, refill reminders, medication schedules) What medication reminder tools do you use? |  | ✓ |  |  |  |  |
| Does your healthcare team (eg. nurses, doctors, pharmacists, etc.) explain to you what your medication(s) are for? |  | ✓ |  |  |  |  |
| Is your health care team (eg. nurses, doctors, pharmacists, etc.) available to answer your questions about your medication(s)? |  | ✓ |  |  |  |  |
| Why do you sometimes need to change the way you take your medication(s)? Please select all that apply to you: ˆ There are too many medication(s).  ˆ I do not feel I need all of these medication(s). ˆ I only take medication(s) when I feel ill. ˆ I have a hard time swallowing some or all of my medication(s) ˆ I cannot get all of my medication(s) from the pharmacy. ˆ I only take medication(s) when my labs are not normal. ˆ My medication(s) are too expensive. ˆ I have a hard time remembering when to take my medication(s). ˆ I am worried about my medications interacting with each other. ˆ I do not like the taste/smell/look of my medication(s). ˆ I do not understand what the medications are for. ˆ I sometimes need to take a break from my medication(s). ˆ I do not change the way I take my medication(s). ˆ I am not able to read/understand the directions for taking my medication(s). ˆ I do not feel the medication(s) result in a visible health benefit. |  | ✓ | ✓ | ✓ | ✓ | ✓ |
| Over the past 7 days, I missed my dose because I could not afford the medication. |  | ✓ |  |  | ✓ | ✓ |
| Over the past 7 days, I missed my dose because I did not want others to see my medications. |  | ✓ |  |  | ✓ | ✓ |
| Over the past 7 days, I missed my dose because there was no one to help me. |  | ✓ |  |  | ✓ | ✓ |
| Over the past 7 days, I missed my dose because treatment was hard on my family. |  | ✓ |  |  | ✓ | ✓ |
| Over the past 7 days, I missed my dose because I could not get answers to my questions about the medication. |  | ✓ | ✓ |  | ✓ | ✓ |
| How often do you run out of medicine? |  | ✓ | ✓ |  | ✓ | ✓ |
| How often do you put off refilling your medicines because they cost too much money? |  | ✓ |  |  |  |  |
| Do you have any difficulty getting your medications on time from the pharmacy? If you answered “sometimes” or “often”, please answer below:  · Is it difficult for you to get to the pharmacy to pick up your medications?  · Is paying for your medications a burden on your finances?  · Do you forget to place refill requests on time? |  | ✓ | ✓ |  |  |  |
| Do you feel that you are NOT receiving the best possible treatment available from your health care provider? |  | ✓ | ✓ |  | ✓ | ✓ |
| Do you feel uncomfortable about taking your medication while you are out with family and friends? |  | ✓ |  |  | ✓ | ✓ |
| In the last month, did you take less of your medication(s) without consulting a physician because of a high medication cost? |  | ✓ |  |  |  |  |
| Do you discontinue these medications because they are not worth of the money you spent on them? |  | ✓ |  |  | ✓ | ✓ |
| Do you forget to take your medication due to your busy schedule, travelling, meeting, events at home, party, marriage, religious celebrations, etc.? |  | ✓ |  |  | ✓ | ✓ |
| Do you find it difficult to buy your medicines because they are expensive? |  | ✓ |  |  |  |  |
| Sometimes, problems at the clinic make it difficult for you to take these medicines every day. Have any of these things been a problem for you:  □ There was no money to purchase medicine (if not offered at AMPATH) □ The medicine was not available in the pharmacy. Which medicine? □ ARVs □ Septrin □ Other (include abx)  □ I finished or ran out of the medicines  □ Other (specify)  □ None of the above |  | ✓ | ✓ |  |  |  |
| It bothers me that others know that I take this medication. |  | ✓ |  |  | ✓ | ✓ |
| I feel comfortable asking my healthcare provider about my medication. |  | ✓ | ✓ |  |  |  |
| My healthcare provider understands when I tell him/her about my preferences in medication taking. |  | ✓ | ✓ |  |  |  |
| My healthcare provider understands when I explain to him/her about my past medication including previous allergic reactions. |  | ✓ | ✓ | ✓ |  |  |
| In the past month, have you missed taking your medications because you: Busy doing other things (e.g., working, trying to survive, getting food?)? |  | ✓ |  |  |  |  |
| In the past month, have you missed taking your medications because you: Didn’t want to bring my pills to social activities (restaurant, friend’s home)? |  | ✓ |  |  |  |  |
| My doctor answers my questions. |  |  | ✓ |  |  |  |
| Do you think you are taking too many medication(s)? |  |  | ✓ | ✓ | ✓ | ✓ |
| Does your pharmacist help you with remembering to take your medication(s)? (example: dosette packs, blister packs, refill reminders, medication schedules) |  |  | ✓ |  |  |  |
| Does your healthcare team (e.g. nurses, doctors, pharmacists, etc.) explain to you what your medication(s) are for? |  |  | ✓ |  |  |  |
| Is your health care team (e.g. nurses, doctors, pharmacists, etc.) available to answer your questions about your medication(s)? |  |  | ✓ |  |  |  |
| Over the past 7 days, I missed my dose because I ran out of medication. |  |  | ✓ | ✓ | ✓ | ✓ |
| Since the last visit have you run out of medicine? |  |  | ✓ |  |  |  |
| I visit the doctor with the recommended frequency. |  |  | ✓ |  |  | ✓ |
| I do not trust health staff; they do not help me. |  |  | ✓ |  | ✓ | ✓ |
| What the doctor tells me, I hang on to. |  |  | ✓ |  | ✓ | ✓ |
| Do you know how to contact our medical staffs when you have a question? |  |  | ✓ |  |  |  |
| In the past month, have you missed taking your medications because you: Didn’t get prescription; ran out of pills? |  |  | ✓ |  |  |  |
| My overall health is excellent. |  |  |  | ✓ | ✓ | ✓ |
| Over the past 4 wk I have never felt blue, downhearted, or depressed. |  |  |  | ✓ | ✓ | ✓ |
| Does taking your medication(s) make you feel upset? |  |  |  | ✓ | ✓ | ✓ |
| Medication can help alleviate my symptoms. |  |  |  | ✓ | ✓ | ✓ |
| Over the past 7 days, I missed my dose because I was feeling too sick to take it. |  |  |  | ✓ | ✓ | ✓ |
| How often do you miss taking you medicine when you feel better? |  |  |  | ✓ |  |  |
| When I take this medication it's like I'm not myself. |  |  |  | ✓ | ✓ | ✓ |
| It's the same whether I take this medication or not. |  |  |  | ✓ | ✓ | ✓ |
| I plan to stop this medication when feeling better. |  |  |  | ✓ | ✓ | ✓ |
| Do you discontinue your medicines due to other medicines that you have to take for your additional disease? |  |  |  | ✓ | ✓ | ✓ |
| During the last month, had there been any occasion when you missed your medicines due to progression of disease and addition of new medicines? |  |  |  | ✓ | ✓ | ✓ |
| Do you know what symptoms will develop when you get worse? |  |  |  | ✓ | ✓ | ✓ |
| Do you know the long-term prognosis of your disease? |  |  |  | ✓ | ✓ | ✓ |
| Do you have your own way to remember to take your medication(s)? |  |  |  |  | ✓ | ✓ |
| Do you feel that you need to know more about what your medication(s) are for? |  |  |  |  | ✓ | ✓ |
| Do you change the way you take any of your medication(s) from what the doctor/nurse/pharmacist recommended? |  |  |  |  | ✓ | ✓ |
| Taking medication is a burden to me. |  |  |  |  | ✓ | ✓ |
| I need to take medication regularly. |  |  |  |  | ✓ | ✓ |
| By staying on medication, I can prevent getting sick and going back to the hospital. |  |  |  |  | ✓ | ✓ |
| Over the past 7 days, I missed my dose because I was out of my routine. |  |  |  |  | ✓ | ✓ |
| Over the past 7 days, I missed my dose because the medication caused side effects. |  |  |  |  | ✓ | ✓ |
| Over the past 7 days, I missed my dose because the medication affected my sex life. |  |  |  |  | ✓ | ✓ |
| Over the past 7 days, I missed my dose because I had other medications to take. |  |  |  |  | ✓ | ✓ |
| Over the past 7 days, I missed my dose because I was afraid the medication would interact with other medication I take, |  |  |  |  | ✓ | ✓ |
| Over the past 7 days, I missed my dose because I forgot. |  |  |  |  | ✓ | ✓ |
| Over the past 7 days, I missed my dose because I did not have my medicines with me. |  |  |  |  | ✓ | ✓ |
| Over the past 7 days, I missed my dose because I was too late with my dose. |  |  |  |  | ✓ | ✓ |
| Over the past 7 days, I missed my dose because I was asleep. |  |  |  |  | ✓ | ✓ |
| Over the past 7 days, I missed my dose because I could not meet the food requirements. |  |  |  |  | ✓ | ✓ |
| Over the past 7 days, I missed my dose because the medication was not working. |  |  |  |  | ✓ | ✓ |
| Do you know the name of your medications? |  |  |  |  | ✓ | ✓ |
| Do you think that you take too many medications? |  |  |  |  | ✓ | ✓ |
| I visit the doctor with the recommended frequency |  |  |  |  | ✓ |  |
| I feel strong enough to fight the disease. |  |  |  |  | ✓ | ✓ |
| I feel anxious when it is medication/insulin time. |  |  |  |  | ✓ | ✓ |
| I believe that my disease will completely cure when my worries or stress is over. |  |  |  |  | ✓ | ✓ |
| I am angry because I have to eat special food and have special needs. |  |  |  |  | ✓ | ✓ |
| I always feel depressed about my future due to my disease. |  |  |  |  | ✓ | ✓ |
| I use a reminder to help me take my medications. |  |  |  |  | ✓ | ✓ |
| I have a routine that works well for taking my medication as prescribed. |  |  |  |  | ✓ | ✓ |
| I keep my medications in a certain place to help me remember to take them. |  |  |  |  | ✓ | ✓ |
| During the last four weeks, how many times were you careless about taking your medication? |  |  |  |  | ✓ | ✓ |
| How often do you miss taking your medicine when you are careless? |  |  |  |  | ✓ | ✓ |
| How often do you change the dose of your medicines to suit your needs (like when you take more or less pill than you’re supposed to)? |  |  |  |  | ✓ | ✓ |
| How often do you forget to take your medicine when you are supposed to take it more than once a day? |  |  |  |  | ✓ | ✓ |
| You have been prescribed medication(s) for your health condition(s) which is to be taken regularly. How would you describe your past experience with taking your medication(s)?  a. I want to be very regular in taking my medication(s), but I am not always good with it due to some challenges.  b. I take my medication(s) regularly (9 out of 10 times).  c. I am not very regular in taking my medication(s) because I feel unwilling. |  |  |  |  | ✓ | ✓ |
| Do you feel that you can take more or less of your medication than the prescribed dose to fit your lifestyle? |  |  |  |  | ✓ | ✓ |
| Do you consider it a burden that you have to take your medications for the rest of your life? |  |  |  |  | ✓ | ✓ |
| Do you have doubts about whether your health condition needs to be treated? |  |  |  |  | ✓ | ✓ |
| Do you have doubts if taking your medication will improve your health condition in the long term? |  |  |  |  | ✓ | ✓ |
| Do you have any other doubts or concerns about taking your medication? |  |  |  |  | ✓ |  |
| Do you feel unsure about how/when to take your medications? |  |  |  |  | ✓ | ✓ |
| Do you forget to place refill requests on time? |  |  |  |  | ✓ | ✓ |
| Do you feel like you don’t get any benefits from taking your medication? |  |  |  |  | ✓ | ✓ |
| Do you worry about what foods or other medications might interact with your medication? |  |  |  |  | ✓ | ✓ |
| I'm ashamed of taking this medication. |  |  |  |  | ✓ | ✓ |
| I find it difficult to take the medication as recommended by the doctor (which pills, at what time…). |  |  |  |  | ✓ | ✓ |
| It bothers me when my medication is changed. |  |  |  |  | ✓ | ✓ |
| My medication has more positive effects than negative ones. |  |  |  |  | ✓ | ✓ |
| When I take this medication, I can think in a clearer manner. |  |  |  |  | ✓ | ✓ |
| Do you have difficulty in remembering to take your medications? |  |  |  |  | ✓ | ✓ |
| Do you find it is a hassle to remember your medications due to medication regime complexity? |  |  |  |  | ✓ | ✓ |
| Do you know the names and usage of your medicines? |  |  |  |  | ✓ | ✓ |
| Do you know the primary role of your medicines? |  |  |  |  | ✓ | ✓ |
| I personally search for and collect information that I want about my medicine. |  |  |  |  | ✓ | ✓ |
| Taking medication is part of my everyday life, just like eating or brushing my teeth. |  |  |  |  | ✓ | ✓ |
| I have stopped taking medication based on my own judgment (not including times when I forgot to take my medication) |  |  |  |  | ✓ | ✓ |
| I report side effects, allergic reactions, or unusual symptoms caused by the medication |  |  |  |  | ✓ | ✓ |
| I sometimes get annoyed that I have to keep taking medicine every day. |  |  |  |  | ✓ | ✓ |
| Do you know why you are taking the medicines? |  |  |  |  | ✓ | ✓ |
| Taking pills everyday is not a big deal. |  |  |  |  | ✓ | ✓ |
| In the past month, have you missed taking your medications because you have to wake up very early to go to work and no time to eat? |  |  |  |  | ✓ | ✓ |
| In the past month, have you missed taking your medications because you didn’t want to bring my pills to social activities (restaurant, friend’s home)? |  |  |  |  | ✓ | ✓ |
| In the past month, have you missed taking your medications because you wanted to have a free day without pills? |  |  |  |  | ✓ | ✓ |
| In the past month, have you missed taking your medications because you forgot? |  |  |  |  | ✓ | ✓ |
| In the past month, have you missed taking your medications because you were busy doing other things (e.g., working, trying to survive, getting food?)? |  |  |  |  | ✓ | ✓ |
| In the past month, have you missed taking your medications because you were too busy at work, school, or home? |  |  |  |  | ✓ | ✓ |
| In the past month, have you missed taking your medications because you lost track of time? |  |  |  |  | ✓ | ✓ |
| In the past month, have you missed taking your medications because you didn’t have a good night sleep? |  |  |  |  | ✓ | ✓ |
| I took a smaller amount of my medication because I was worried about its side effects. |  |  |  |  | ✓ | ✓ |
| Do you think your treatment will last too long? |  |  |  |  | ✓ | ✓ |
