# Supplementary material for: Development of an Item Bank to Measure Medication Adherence: Systematic Review
Source: J Med Internet Res. 2020 Oct 8;22(10):e19089. doi: 10.2196/19089 (PMC7582150; doi:10.2196/19089)
Supplement: Multimedia Appendix 4 [file jmir_v22i10e19089_app4.docx]

| No. | Item | Instrument of Origin |
| --- | --- | --- |
| Extent of Adherence | | |
|  | Do you remember to take your medication(s)? | Medication Adherence Survey for Hemodialysis Patients |
|  | Do you change the way you take any of your medication(s) from what the doctor/nurse/pharmacist recommended? | Medication Adherence Survey for Hemodialysis Patients |
|  | If you change the way you take your medication(s) what do you do differently? Please select all that apply to you: ˆ I change how many times a day I take them. ˆ I skip some of the doses. ˆ I stop taking the medication(s). ˆ I do not take the medication(s) with/without food like the doctor/nurse/pharmacist suggested because they are too hard to take with/without food. ˆ I find some of the medication(s) too hard to take at the same time as others like the doctor/nurse/pharmacist suggested. ˆ I change some of the medication(s) that were prescribed to something I feel works the same. ˆ I do not change the way I take my medication(s). | Medication Adherence Survey for Hemodialysis Patients |
|  | I follow the doctor's instruction to take medication. | BMAS |
|  | I forget to take my medication. | BMAS |
|  | I adjust the medication and dosage based on my condition. | BMAS |
|  | If I have the side effect of the medication, I will stop taking it. | BMAS |
|  | I take medication only when I am sick. | BMAS |
|  | Over the past 7 days, I missed my medicine. | DOSE-Nonadherence |
|  | Over the past 7 days, I skipped a dose of my medicine. | DOSE-Nonadherence |
|  | Over the past 7 days, I did not take a dose of my medicine. | DOSE-Nonadherence |
|  | This morning did you forget to take your medicine? | Self-Assessment Tool to Measure Imatinib Adherence in Patients with Chronic Myeloid Leukemia |
|  | Do you ever take your medicine too late in comparison with usual time? | Self-Assessment Tool to Measure Imatinib Adherence in Patients with Chronic Myeloid Leukemia |
|  | Sometimes if you feel worse when you take your medicine, do you stop taking it? | Self-Assessment Tool to Measure Imatinib Adherence in Patients with Chronic Myeloid Leukemia |
|  | Do you ever not take your medicine because you forgot to do so? | Self-Assessment Tool to Measure Imatinib Adherence in Patients with Chronic Myeloid Leukemia |
|  | Do you ever miss doses of your medicine when you feel sick? | Self-Assessment Tool to Measure Imatinib Adherence in Patients with Chronic Myeloid Leukemia |
|  | Does a change in your daily routine modify the way you take your medicine? | Self-Assessment Tool to Measure Imatinib Adherence in Patients with Chronic Myeloid Leukemia |
|  | Do you sometimes skip doses of your medicine when you feel better? | Self-Assessment Tool to Measure Imatinib Adherence in Patients with Chronic Myeloid Leukemia |
|  | I take my pills at the same time each day. | Patient-Reported Measures Assessing Adherence Behaviors and Barriers in Patients Living with HIV |
|  | In the past 30 days, I was late by one hour or more in taking my medication. | Patient-Reported Measures Assessing Adherence Behaviors and Barriers in Patients Living with HIV |
|  | In the past 30 days, I skipped taking some or all of my medications ON PURPOSE. | Patient-Reported Measures Assessing Adherence Behaviors and Barriers in Patients Living with HIV |
|  | How much of each medicine have you missed taking in the last 7 days? | DAMS |
|  | How much extra of each medicine did you take in the last 7 days? | DAMS |
|  | Do you ever forget to take your medicine? | SMAQ |
|  | Are you careless at times about taking your medicine? | SMAQ |
|  | Thinking about the last week. How often have you not taken your medicine? | SMAQ |
|  | Over the past 3 months, how many days have you not taken any medicine at all? | SMAQ |
|  | How often do you skip a dose of your medicine before you go to the doctor? | ARMS |
|  | How often do you forget to get prescriptions filled? | ARMS |
|  | How often do you forget to take your medicine? | ARMS |
|  | How often do you decide not to take your medicine? | ARMS |
|  | How often do you miss taking your medicine when you feel better? | ARMS |
|  | How often do you miss taking your medicine when you feel sick? | ARMS |
|  | How often do you plan ahead and refill your medicines before they run out? | ARMS |
|  | In the last month, how often did you forget to take your medication(s)? | IADMAS |
|  | In the last month, how often did you intend to take your medication(s) in doses different to what has been prescribed? | IADMAS |
|  | In the last month, how often did you intend to take your medication(s) in a time different to what has been prescribed? | IADMAS |
|  | In the last month, did you take your medication(s) with you when you are away from home (e.g., traveling or visiting relatives)? | IADMAS |
|  | In the last month, did you stop taking your medication(s) without consulting a physician because of medication side effects? | IADMAS |
|  | In the last month, did you take less of your medication (s) without consulting a physician because you feel better? | IADMAS |
|  | During sick days (e.g., flu, and diarrhea), did you take less of your medication (s) without consulting a physician due to reduced appetite? | IADMAS |
|  | In the last month, did you take less of your medication (s) without consulting a physician because of a high medication cost? | IADMAS |
|  | Do you stop taking medications without informing the doctor? | GMAS |
|  | Do you alter medication regimen, dose and frequency by yourself? | GMAS |
|  | Over the past 3 weeks, I have taken the prescribed daily dosage of my medication. | 12-Item Medication Adherence Scale for Patients with Chronic Disease |
|  | I accept the necessity of taking medication in the prescribed manner to treat my illness. | 12-Item Medication Adherence Scale for Patients with Chronic Disease |
|  | I have stopped taking medication based on my own judgment (not including times when I forgot to take my medication). | 12-Item Medication Adherence Scale for Patients with Chronic Disease |
|  | During the last four weeks, how many times did you forget to take your medication? | AAS |
|  | During the last four weeks, how many times, when you felt better, did you stop taking your medication? | AAS |
|  | During the last four weeks, how many times, when you felt worse, did stop taking your medication? | AAS |
|  | Do you ever have problems keeping time with the medicines? When? | ICAMP |
|  | Some families tell us that their child worries them or makes it difficult to give them the medicines. Have you not taken medicines for any of these reasons: □ I do not know why taking am taking the medicines or keeps asking questions about the medicines □ I forgot to take medicine □ I felt ill or was vomiting  □ I was playing or at school or work  □ I refused to take medicine  □ I have problems with 1 formulation (tablets, liquids)  □ I find medicines too bitter □ I can’t take without food □ None of the above □ Other (specify) | ICAMP |
|  | In the past week,  a. On how many days did you miss at least one dose? b. On how many days did you take a dose more than an hour late?  c. How many extra doses or syringes of medicine did you take? | ICAMP |
|  | How many doses of medicine did you miss in the last month? | ICAMP |
|  | I skipped a dose of my medication because I was worried about its side effects. | MEDS |
|  | I skipped a dose of my medication because I was having side effects. | MEDS |
|  | I took a smaller amount of my medication because I was worried about its side effects. | MEDS |
|  | I took a smaller amount of my medication because I was having side effects. | MEDS |
|  | I skipped a dose of my medication because I was worried about getting addicted to it. | MEDS |
|  | I took a smaller amount of my medication because I was worried about getting addicted to it. | MEDS |
|  | I skipped a dose of my medication because I was worried about costs. | MEDS |
|  | I took a smaller amount of my medication because I was worried about costs. | MEDS |
|  | I skipped a dose of my medication because I was feeling better. | MEDS |
|  | I skipped a dose of my medication because I did not need it. | MEDS |
|  | I took a smaller amount of my medication because I was feeling better. | MEDS |
|  | I took a smaller amount of my medication because I did not need it. | MEDS |
|  | I forgot to take a dose of my medication. | MEDS |
|  | I missed a dose of my medication by mistake. | MEDS |
|  | I missed a dose of my medication because I did not get it refilled before I ran out. | MEDS |
|  | I missed a dose of my medication because I forgot to take it with me. | MEDS |
|  | Number of daily doses and number of doses missed over the last 28 days. | Every Visit Adherence Questionnaire |
|  | Do you just forget to take medicines some of the time? | Risk of Nonadherence to Antibiotic Treatment Questionnaire |
|  | Have you taken a medicine more or less often than prescribed? | Risk of Nonadherence to Antibiotic Treatment Questionnaire |
|  | Have you skipped or stopped taking a medicine because you did not think it was working? | Risk of Nonadherence to Antibiotic Treatment Questionnaire |
|  | Have you not had medicine with you when it was time to take it? | Risk of Nonadherence to Antibiotic Treatment Questionnaire |
|  | Are you careless at times about taking your medicine? | Risk of Nonadherence to Antibiotic Treatment Questionnaire |
|  | Did you ever take the medication less frequently or at a smaller dose than was prescribed, or stopped the medication on your own? | TAS-P |
| Reasons for Non-adherence (Social and Economic Factors) | | |
|  | Does taking your medication(s) affect the way you live your life? | Medication Adherence Survey for Hemodialysis Patients |
|  | Does someone (spouse/family/friends) help you remember to take your medication(s)? | Medication Adherence Survey for Hemodialysis Patients |
|  | Does your pharmacist help you with remembering to take your medication(s)? (example: dosette packs, blister packs, refill reminders, medication schedules) What medication reminder tools do you use? | Medication Adherence Survey for Hemodialysis Patients |
|  | Does your healthcare team (eg. nurses, doctors, pharmacists, etc.) explain to you what your medication(s) are for? | Medication Adherence Survey for Hemodialysis Patients |
|  | Is your health care team (eg. nurses, doctors, pharmacists, etc.) available to answer your questions about your medication(s)? | Medication Adherence Survey for Hemodialysis Patients |
|  | Why do you sometimes need to change the way you take your medication(s)? Please select all that apply to you: ˆ There are too many medication(s).  ˆ I do not feel I need all of these medication(s). ˆ I only take medication(s) when I feel ill. ˆ I have a hard time swallowing some or all of my medication(s) ˆ I cannot get all of my medication(s) from the pharmacy. ˆ I only take medication(s) when my labs are not normal. ˆ My medication(s) are too expensive. ˆ I have a hard time remembering when to take my medication(s). ˆ I am worried about my medications interacting with each other. ˆ I do not like the taste/smell/look of my medication(s). ˆ I do not understand what the medications are for. ˆ I sometimes need to take a break from my medication(s). ˆ I do not change the way I take my medication(s). ˆ I am not able to read/understand the directions for taking my medication(s). ˆ I do not feel the medication(s) result in a visible health benefit. | Medication Adherence Survey for Hemodialysis Patients |
|  | Over the past 7 days, I missed my dose because I could not afford the medication. | DOSE-Nonadherence |
|  | Over the past 7 days, I missed my dose because I did not want others to see my medications. | DOSE-Nonadherence |
|  | Over the past 7 days, I missed my dose because there was no one to help me. | DOSE-Nonadherence |
|  | Over the past 7 days, I missed my dose because treatment was hard on my family. | DOSE-Nonadherence |
|  | Over the past 7 days, I missed my dose because I could not get answers to my questions about the medication. | DOSE-Nonadherence |
|  | How often do you run out of medicine? | ARMS |
|  | How often do you put off refilling your medicines because they cost too much money? | ARMS |
|  | How often do you forget to get prescriptions filled? | ARMS |
|  | How often do you plan ahead and refill your medicines before they run out? | ARMS |
|  | Do you have any difficulty getting your medications on time from the pharmacy? If you answered “sometimes” or “often”, please answer below:  · Is it difficult for you to get to the pharmacy to pick up your medications?  · Is paying for your medications a burden on your finances?  · Do you forget to place refill requests on time? | M-DRAW |
|  | Do you feel that you are NOT receiving the best possible treatment available from your health care provider? | M-DRAW |
|  | Do you feel uncomfortable about taking your medication while you are out with family and friends? | M-DRAW |
|  | In the last month, did you take less of your medication(s) without consulting a physician because of a high medication cost? | IADMAS |
|  | Do you discontinue these medications because they are not worth of the money you spent on them? | GMAS |
|  | Do you forget to take your medication due to your busy schedule, travelling, meeting, events at home, party, marriage, religious celebrations, etc.? | GMAS |
|  | Do you find it difficult to buy your medicines because they are expensive? | GMAS |
|  | Sometimes, problems at the clinic make it difficult for you to take these medicines every day. Have any of these things been a problem for you:  □ There was no money to purchase medicine (if not offered at AMPATH) □ The medicine was not available in the pharmacy. Which medicine? □ ARVs □ Septrin □ Other (include abx)  □ I finished or ran out of the medicines  □ Other (specify)  □ None of the above | ICAMP |
|  | It bothers me that others know that I take this medication. | AMBAS |
|  | I feel comfortable asking my healthcare provider about my medication. | 12-Item Medication Adherence Scale for Patients with Chronic Disease |
|  | My healthcare provider understands when I tell him/her about my preferences in medication taking. | 12-Item Medication Adherence Scale for Patients with Chronic Disease |
|  | My healthcare provider understands when I explain to him/her about my past medication including previous allergic reactions. | 12-Item Medication Adherence Scale for Patients with Chronic Disease |
|  | In the past month, have you missed taking your medications because you: Busy doing other things (e.g., working, trying to survive, getting food?)? | IRT-30 |
|  | In the past month, have you missed taking your medications because you: Didn’t want to bring my pills to social activities (restaurant, friend’s home)? | IRT-30 |
|  | I skipped a dose of my medication because I was worried about costs. | MEDS |
|  | I took a smaller amount of my medication because I was worried about costs. | MEDS |
|  | I missed a dose of my medication because I did not get it refilled before I ran out. | MEDS |
| Reasons for Non-adherence (Health Care Team and System-related Factors) | | |
|  | My doctor answers my questions. | Reduced GTCAT |
|  | Do you think you are taking too many medication(s)? | Medication Adherence Survey for Hemodialysis Patients |
|  | Does someone (spouse/family/friends) help you remember to take your medication(s)? | Medication Adherence Survey for Hemodialysis Patients |
|  | Why do you sometimes need to change the way you take your medication(s)? Please select all that apply to you: ˆ There are too many medication(s).  ˆ I do not feel I need all of these medication(s). ˆ I only take medication(s) when I feel ill. ˆ I have a hard time swallowing some or all of my medication(s) ˆ I cannot get all of my medication(s) from the pharmacy. ˆ I only take medication(s) when my labs are not normal. ˆ My medication(s) are too expensive. ˆ I have a hard time remembering when to take my medication(s). ˆ I am worried about my medications interacting with each other. ˆ I do not like the taste/smell/look of my medication(s). ˆ I do not understand what the medications are for. ˆ I sometimes need to take a break from my medication(s). ˆ I do not change the way I take my medication(s). ˆ I am not able to read/understand the directions for taking my medication(s). ˆ I do not feel the medication(s) result in a visible health benefit. | Medication Adherence Survey for Hemodialysis Patients |
|  | Does your pharmacist help you with remembering to take your medication(s)? (example: dosette packs, blister packs, refill reminders, medication schedules) | Medication Adherence Survey for Hemodialysis Patients |
|  | Does your healthcare team (e.g. nurses, doctors, pharmacists, etc.) explain to you what your medication(s) are for? | Medication Adherence Survey for Hemodialysis Patients |
|  | Is your health care team (e.g. nurses, doctors, pharmacists, etc.) available to answer your questions about your medication(s)? | Medication Adherence Survey for Hemodialysis Patients |
|  | Over the past 7 days, I missed my dose because I ran out of medication. | DOSE-Nonadherence |
|  | Over the past 7 days, I missed my dose because I could not get answers to my questions about the medication. | DOSE-Nonadherence |
|  | Since the last visit have you run out of medicine? | Self-Assessment Tool to Measure Imatinib Adherence in Patients with Chronic Myeloid Leukemia |
|  | I visit the doctor with the recommended frequency. | Assessment Scale for Treatment Compliance in Type 2 Diabetes Mellitus |
|  | I do not trust health staff; they do not help me. | Assessment Scale for Treatment Compliance in Type 2 Diabetes Mellitus |
|  | What the doctor tells me, I hang on to. | CQR5 |
|  | How often do you forget to get prescriptions filled? | ARMS |
|  | How often do you run out of medicine? | ARMS |
|  | How often do you plan ahead and refill your medicines before they run out? | ARMS |
|  | Do you feel that you are NOT receiving the best possible treatment available from your health care provider? | M-DRAW |
|  | Do you have any difficulty getting your medications on time from the pharmacy? If you answered “sometimes” or “often”, please answer below:  · Is it difficult for you to get to the pharmacy to pick up your medications?  · Is paying for your medications a burden on your finances?  · Do you forget to place refill requests on time? | M-DRAW |
|  | I feel comfortable asking my healthcare provider about my medication. | 12-Item Medication Adherence Scale for Patients with Chronic Disease |
|  | My healthcare provider understands when I tell him/her about my preferences in medication taking. | 12-Item Medication Adherence Scale for Patients with Chronic Disease |
|  | My healthcare provider understands when I explain to him/her about my past medication including previous allergic reactions. | 12-Item Medication Adherence Scale for Patients with Chronic Disease |
|  | Sometimes, problems at the clinic make it difficult for you to take these medicines every day. Have any of these things been a problem for you:  □ There was no money to purchase medicine (if not offered at AMPATH) □ The medicine was not available in the pharmacy. Which medicine? □ ARVs □ Septrin □ Other (include abx)  □ I finished or ran out of the medicines  □ Other (specify)  □ None of the above | ICAMP |
|  | Do you know how to contact our medical staffs when you have a question? | Chinese and Western Medication Adherence Scale in Chronic Kidney Disease |
|  | In the past month, have you missed taking your medications because you: Didn’t get prescription; ran out of pills? | IRT-30 |
| Reasons for Non-adherence (Condition-related Factors) | | |
|  | My overall health is excellent | Reduced GTCAT |
|  | Over the past 4 wk I have never felt blue, downhearted, or depressed | Reduced GTCAT |
|  | Does taking your medication(s) make you feel upset? | Medication Adherence Survey for Hemodialysis Patients |
|  | Do you think you are taking too many medication(s)? | Medication Adherence Survey for Hemodialysis Patients |
|  | If you change the way you take your medication(s) what do you do differently? Please select all that apply to you: ˆ I change how many times a day I take them. ˆ I skip some of the doses. ˆ I stop taking the medication(s). ˆ I do not take the medication(s) with/without food like the doctor/nurse/pharmacist suggested because they are too hard to take with/without food. ˆ I find some of the medication(s) too hard to take at the same time as others like the doctor/nurse/pharmacist suggested. ˆ I change some of the medication(s) that were prescribed to something I feel works the same. ˆ I do not change the way I take my medication(s). | Medication Adherence Survey for Hemodialysis Patients |
|  | Why do you sometimes need to change the way you take your medication(s)? Please select all that apply to you: ˆ There are too many medication(s).  ˆ I do not feel I need all of these medication(s). ˆ I only take medication(s) when I feel ill. ˆ I have a hard time swallowing some or all of my medication(s) ˆ I cannot get all of my medication(s) from the pharmacy. ˆ I only take medication(s) when my labs are not normal. ˆ My medication(s) are too expensive. ˆ I have a hard time remembering when to take my medication(s). ˆ I am worried about my medications interacting with each other. ˆ I do not like the taste/smell/look of my medication(s). ˆ I do not understand what the medications are for. ˆ I sometimes need to take a break from my medication(s). ˆ I do not change the way I take my medication(s). ˆ I am not able to read/understand the directions for taking my medication(s). ˆ I do not feel the medication(s) result in a visible health benefit. | Medication Adherence Survey for Hemodialysis Patients |
|  | Medication can help alleviate my symptoms. | BMAS |
|  | I adjust the medication and dosage based on my condition. | BMAS |
|  | I take medication only when I am sick. | BMAS |
|  | Over the past 7 days, I missed my dose because I was feeling too sick to take it. | DOSE-Nonadherence |
|  | Over the past 7 days, I missed my dose because I ran out of medication. | DOSE-Nonadherence |
|  | Sometimes if you feel worse when you take your medicine, do you stop taking it? | Self-Assessment Tool to Measure Imatinib Adherence in Patients with Chronic Myeloid Leukemia |
|  | Do you sometimes skip doses of your medicine when you feel better? | Self-Assessment Tool to Measure Imatinib Adherence in Patients with Chronic Myeloid Leukemia |
|  | Do you ever miss doses of your medicine when you feel sick? | Self-Assessment Tool to Measure Imatinib Adherence in Patients with Chronic Myeloid Leukemia |
|  | During the last four weeks, how many times, when you felt better, did you stop taking your medication? | AAS |
|  | During the last four weeks, how many times, when you felt worse, did stop taking your medication? | AAS |
|  | How often do you miss taking you medicine when you feel better? | ARMS |
|  | How often do you forget to take your medicine? | ARMS |
|  | How often do you miss taking your medicine when you feel sick? | ARMS |
|  | How often do you plan ahead and refill your medicines before they run out? | ARMS |
|  | When I take this medication it's like I'm not myself. | AMBAS |
|  | It's the same whether I take this medication or not. | AMBAS |
|  | I plan to stop this medication when feeling better. | AMBAS |
|  | In the last month, did you take less of your medication (s) without consulting a physician because you feel better? | IADMAS |
|  | During sick days (e.g., flu, and diarrhea), did you take less of your medication (s) without consulting a physician due to reduced appetite? | IADMAS |
|  | Do you discontinue your medicines due to other medicines that you have to take for your additional disease? | GMAS |
|  | During the last month, had there been any occasion when you missed your medicines due to progression of disease and addition of new medicines? | GMAS |
|  | Do you know what symptoms will develop when you get worse? | Chinese and Western Medication Adherence Scale in Chronic Kidney Disease |
|  | Do you know the long-term prognosis of your disease? | Chinese and Western Medication Adherence Scale in Chronic Kidney Disease |
|  | I have stopped taking medication based on my own judgment (not including times when I forgot to take my medication). | 12-Item Medication Adherence Scale for Patients with Chronic Disease |
|  | My healthcare provider understands when I explain to him/her about my past medication including previous allergic reactions. | 12-Item Medication Adherence Scale for Patients with Chronic Disease |
|  | Some families tell us that their child worries them or makes it difficult to give them the medicines. Have you not taken medicines for any of these reasons:  □ I do not know why taking am taking the medicines or keeps asking questions about the medicines  □ I forgot to take medicine □ I felt ill or was vomiting  □ I was playing or at school or work  □ I refused to take medicine  □ I have problems with 1 formulation (tablets, liquids)  □ I find medicines too bitter □ I can’t take without food □ None of the above □ Other (specify) | ICAMP |
|  | I skipped a dose of my medication because I was feeling better. | MEDS |
|  | I skipped a dose of my medication because I did not need it. | MEDS |
|  | I took a smaller amount of my medication because I was feeling better. | MEDS |
|  | I took a smaller amount of my medication because I did not need it. | MEDS |
|  | Have you taken a medicine more or less often than prescribed? | Risk of Nonadherence to Antibiotic Treatment Questionnaire |
| Reasons for Non-adherence (Therapy-related Factors) | | |
|  | Does taking your medication(s) make you feel upset? | Medication Adherence Survey for Hemodialysis Patients |
|  | Does taking your medication(s) affect the way you live your life? | Medication Adherence Survey for Hemodialysis Patients |
|  | Do you remember to take your medication(s)? | Medication Adherence Survey for Hemodialysis Patients |
|  | Do you think you are taking too many medication(s)? | Medication Adherence Survey for Hemodialysis Patients |
|  | Do you have your own way to remember to take your medication(s)? | Medication Adherence Survey for Hemodialysis Patients |
|  | Does your pharmacist help you with remembering to take your medication(s)? (example: dosette packs, blister packs, refill reminders, medication schedules) What medication reminder tools do you use? | Medication Adherence Survey for Hemodialysis Patients |
|  | Do you change the way you take any of your medication(s) from what the doctor/nurse/pharmacist recommended? | Medication Adherence Survey for Hemodialysis Patients |
|  | If you change the way you take your medication(s) what do you do differently? Please select all that apply to you: ˆ I change how many times a day I take them. ˆ I skip some of the doses. ˆ I stop taking the medication(s). ˆ I do not take the medication(s) with/without food like the doctor/nurse/pharmacist suggested because they are too hard to take with/without food. ˆ I find some of the medication(s) too hard to take at the same time as others like the doctor/nurse/pharmacist suggested. ˆ I change some of the medication(s) that were prescribed to something I feel works the same. ˆ I do not change the way I take my medication(s). | Medication Adherence Survey for Hemodialysis Patients |
|  | Why do you sometimes need to change the way you take your medication(s)? Please select all that apply to you: ˆ There are too many medication(s).  ˆ I do not feel I need all of these medication(s). ˆ I only take medication(s) when I feel ill. ˆ I have a hard time swallowing some or all of my medication(s) ˆ I cannot get all of my medication(s) from the pharmacy. ˆ I only take medication(s) when my labs are not normal. ˆ My medication(s) are too expensive. ˆ I have a hard time remembering when to take my medication(s). ˆ I am worried about my medications interacting with each other. ˆ I do not like the taste/smell/look of my medication(s). ˆ I do not understand what the medications are for. ˆ I sometimes need to take a break from my medication(s). ˆ I do not change the way I take my medication(s). ˆ I am not able to read/understand the directions for taking my medication(s). ˆ I do not feel the medication(s) result in a visible health benefit. | Medication Adherence Survey for Hemodialysis Patients |
|  | Medication makes me feel tired and sluggish. | BMAS |
|  | Taking medication is a burden to me. | BMAS |
|  | I forget to take my medication. | BMAS |
|  | I adjust the medication and dosage based on my condition. | BMAS |
|  | If I have the side effect of the medication, I will stop taking it. | BMAS |
|  | Over the past 7 days, I missed my dose because I was out of my routine. | DOSE-Nonadherence |
|  | Over the past 7 days, I missed my dose because the medication caused side effects. | DOSE-Nonadherence |
|  | Over the past 7 days, I missed my dose because I could not meet the food requirements. | DOSE-Nonadherence |
|  | Over the past 7 days, I missed my dose because I did not have my medicines with me | DOSE-Nonadherence |
|  | Over the past 7 days, I missed my dose because I could not afford the medication | DOSE-Nonadherence |
|  | Over the past 7 days, I missed my dose because the medication was not working. | DOSE-Nonadherence |
|  | Over the past 7 days, I missed my dose because I did not want others to see my medications | DOSE-Nonadherence |
|  | Over the past 7 days, I missed my dose because the medication affected my sex life. | DOSE-Nonadherence |
|  | Over the past 7 days, I missed my dose because I had other medications to take. | DOSE-Nonadherence |
|  | Over the past 7 days, I missed my dose because I was too late with my dose. | DOSE-Nonadherence |
|  | Over the past 7 days, I missed my dose because I was asleep. | DOSE-Nonadherence |
|  | Over the past 7 days, I missed my dose because treatment was hard on my family. | DOSE-Nonadherence |
|  | Over the past 7 days, I missed my dose because there was no one to help me. | DOSE-Nonadherence |
|  | Over the past 7 days, I missed my dose because I was feeling too sick to take it. | DOSE-Nonadherence |
|  | Over the past 7 days, I missed my dose because I could not get answers to my questions about the medication. | DOSE-Nonadherence |
|  | Over the past 7 days, I missed my dose because I ran out of medication. | DOSE-Nonadherence |
|  | Over the past 7 days, I missed my dose because I was afraid the medication would interact with other medication I take. | DOSE-Nonadherence |
|  | Do you ever take your medicine too late in comparison with usual time? | Self-Assessment Tool to Measure Imatinib Adherence in Patients with Chronic Myeloid Leukemia |
|  | Sometimes if you feel worse when you take your medicine, do you stop taking it? | Self-Assessment Tool to Measure Imatinib Adherence in Patients with Chronic Myeloid Leukemia |
|  | Do you think that you take too many medications? | Self-Assessment Tool to Measure Imatinib Adherence in Patients with Chronic Myeloid Leukemia |
|  | Do you ever not take your medicine because you forgot to do so? | Self-Assessment Tool to Measure Imatinib Adherence in Patients with Chronic Myeloid Leukemia |
|  | Do you ever miss doses of your medicine when you feel sick? | Self-Assessment Tool to Measure Imatinib Adherence in Patients with Chronic Myeloid Leukemia |
|  | Does a change in your daily routine modify the way you take your medicine? | Self-Assessment Tool to Measure Imatinib Adherence in Patients with Chronic Myeloid Leukemia |
|  | Do you sometimes skip doses of your medicine when you feel better? | Self-Assessment Tool to Measure Imatinib Adherence in Patients with Chronic Myeloid Leukemia |
|  | I take my pills at the same time each day. | Patient-Reported Measures Assessing Adherence Behaviors and Barriers in Patients Living with HIV |
|  | I have a routine that works well for taking my medication as prescribed. | Patient-Reported Measures Assessing Adherence Behaviors and Barriers in Patients Living with HIV |
|  | I use a reminder to help me take my medications. | Patient-Reported Measures Assessing Adherence Behaviors and Barriers in Patients Living with HIV |
|  | I keep my medications in a certain place to help me remember to take them. | Patient-Reported Measures Assessing Adherence Behaviors and Barriers in Patients Living with HIV |
|  | During the last four weeks, how many times were you careless about taking your medication? | AAS |
|  | During the last four weeks, how many times, when you felt better, did you stop taking your medication? | AAS |
|  | Do you have difficulty keeping track of all your medication schedules throughout the day? | M-DRAW |
|  | Do you feel like you don’t get any benefits from taking your medication? | M-DRAW |
|  | You have been prescribed medication(s) for your health condition(s) which is to be taken regularly. How would you describe your past experience with taking your medication(s)?  a. I want to be very regular in taking my medication(s), but I am not always good with it due to some challenges.  b. I take my medication(s) regularly (9 out of 10 times).  c. I am not very regular in taking my medication(s) because I feel unwilling. | M-DRAW |
|  | Do your medications give you side effects that make you NOT want to take it? | M-DRAW |
|  | Do you worry about what foods or other medications might interact with your medication? | M-DRAW |
|  | Do you feel that you can take more or less of your medication than the prescribed dose to fit your lifestyle? | M-DRAW |
|  | Do you have doubts if taking your medication will improve your health condition in the long term? | M-DRAW |
|  | Thinking of the medicines your doctor has prescribed: A. Which medicines have you been asked to take? B. How MUCH of these MEDICINES have you been asked to take each DAY? | DAMS |
|  | Thinking of the medicines your doctor has prescribed: A. How many tablets and capsules have you been asked to take each day? B. How many spoonfuls of medicine have you been asked to take each day? C. How many puffs of inhalers have you been asked to take each day? D. How many creams have you been asked to use each day? E. How many injections have you been asked to have each day? F. Have you been asked to use any other type of medication? If so, how much? | DAMS |
|  | People often take more of their medicine than has been prescribed. Thinking of the last 7 days: A. How MUCH EXTRA of each medicine did you take in the last 7 DAYS? | DAMS |
|  | THINKING OF THE EXTRA MEDICINE YOU HAVE TAKEN IN THE LAST 7 DAYS, which of the following statements best describe what happened (you can choose more than one option)? A. I decided to take more B. I accidentally took more C. Other (please specify) | DAMS |
|  | When I take this medication it's like I'm not myself. | AMBAS |
|  | I find it difficult to take the medication as recommended by the doctor. | AMBAS |
|  | I find it difficult to take the medication as recommended by the doctor (which pills, at what time…). | AMBAS |
|  | It bothers me when my medication is changed. | AMBAS |
|  | I plan to stop this medication when feeling better. | AMBAS |
|  | My medication has more positive effects than negative ones. | AMBAS |
|  | When I take this medication, I can think in a clearer manner. | AMBAS |
|  | This medication does not appear to have any positive effects. | AMBAS |
|  | Taking this medication prevents relapses. | AMBAS |
|  | Do you have difficulty in remembering to take your medications? | GMAS |
|  | Do you forget to take your medication due to your busy schedule, travelling, meeting, events at home, party, marriage, religious celebrations, etc.? | GMAS |
|  | Do you stop taking medications without informing the doctor? | GMAS |
|  | Do you discontinue your medicines due to other medicines that you have to take for your additional disease? | GMAS |
|  | Do you alter medication regimen, dose and frequency by yourself? | GMAS |
|  | Do you discontinue these medications because they are not worth of the money you spent on them? | GMAS |
|  | Do you find it is a hassle to remember your medications due to medication regime complexity? | GMAS |
|  | During the last month, had there been any occasion when you missed your medicines due to progression of disease and addition of new medicines? | GMAS |
|  | Overall, how satisfied are you with how things have been with your treatment during the past 4 weeks (28 days)? | MS-TAQ |
|  | In the last month, how often did you forget to take your medication(s)? | IADMAS |
|  | In the last month, did you take your medication(s) with you when you are away from home (e.g., traveling or visiting relatives)? | IADMAS |
|  | In the last month, did you stop taking your medication(s) without consulting a physician because of medication side effects? | IADMAS |
|  | In the last month, did you take less of your medication (s) without consulting a physician because you feel better? | IADMAS |
|  | During sick days (e.g., flu, and diarrhea), did you take less of your medication (s) without consulting a physician due to reduced appetite? | IADMAS |
|  | I have stopped taking medication based on my own judgment (not including times when I forgot to take my medication). | 12-Item Medication Adherence Scale for Patients with Chronic Disease |
|  | My healthcare provider understands when I explain to him/her about my past medication including previous allergic reactions. | 12-Item Medication Adherence Scale for Patients with Chronic Disease |
|  | I understand both the effects and the side effects of my medication. | 12-Item Medication Adherence Scale for Patients with Chronic Disease |
|  | I report side effects, allergic reactions, or unusual symptoms caused by the medication. | 12-Item Medication Adherence Scale for Patients with Chronic Disease |
|  | I sometimes get annoyed that I have to keep taking medicine every day. | 12-Item Medication Adherence Scale for Patients with Chronic Disease |
|  | Do you ever have problems keeping time with the medicines? When? | ICAMP |
|  | Do you ever have problems with taking the medicines? What problems do you have? (explain) | ICAMP |
|  | Some families tell us that their child worries them or makes it difficult to give them the medicines. Have you not taken medicines for any of these reasons:  □ I do not know why taking am taking the medicines or keeps asking questions about the medicines  □ I forgot to take medicine □ I felt ill or was vomiting  □ I was playing or at school or work  □ I refused to take medicine  □ I have problems with 1 formulation (tablets, liquids)  □ I find medicines too bitter □ I can’t take without food □ None of the above □ Other (specify) | ICAMP |
|  | In the past week,  a. On how many days did you miss at least one dose? b. On how many days did you take a dose more than an hour late?  c. How many extra doses or syringes of medicine did you take? | ICAMP |
|  | How many doses of medicine did you miss in the last month? | ICAMP |
|  | In the past month, have you missed taking your medications because you forgot? | IRT-30 |
|  | I skipped a dose of my medication because I was worried about its side effects. | MEDS |
|  | I skipped a dose of my medication because I was having side effects. | MEDS |
|  | I took a smaller amount of my medication because I was worried about its side effects. | MEDS |
|  | I took a smaller amount of my medication because I was having side effects. | MEDS |
|  | I skipped a dose of my medication because I was worried about getting addicted to it. | MEDS |
|  | I took a smaller amount of my medication because I was worried about getting addicted to it. | MEDS |
|  | I skipped a dose of my medication because I was worried about costs. | MEDS |
|  | I took a smaller amount of my medication because I was worried about costs. | MEDS |
|  | I skipped a dose of my medication because I was feeling better. | MEDS |
|  | I took a smaller amount of my medication because I was feeling better. | MEDS |
|  | I missed a dose of my medication because I forgot to take it with me. | MEDS |
|  | How often do you forget to take your medicine? | ARMS |
|  | How often do you decide not to take your medicine? | ARMS |
|  | How often do you forget to get prescriptions filled? | ARMS |
|  | How often do you run out of medicine? | ARMS |
|  | How often do you miss taking your medicine when you feel better? | ARMS |
|  | How often do you miss taking your medicine when you feel sick? | ARMS |
|  | How often do you plan ahead and refill your medicines before they run out? | ARMS |
|  | Is taking medicines more than once a day inconvenient? | Risk of Nonadherence to Antibiotic Treatment Questionnaire |
|  | Do you think your treatment will last too long? | Risk of Nonadherence to Antibiotic Treatment Questionnaire |
|  | Do you just forget to take medicines some of the time? | Risk of Nonadherence to Antibiotic Treatment Questionnaire |
|  | Have you taken a medicine more or less often than prescribed? | Risk of Nonadherence to Antibiotic Treatment Questionnaire |
|  | Have you skipped or stopped taking a medicine because you did not think it was working? | Risk of Nonadherence to Antibiotic Treatment Questionnaire |
|  | Have you not had medicine with you when it was time to take it? | Risk of Nonadherence to Antibiotic Treatment Questionnaire |
|  | Are you careless at times about taking your medicine? | Risk of Nonadherence to Antibiotic Treatment Questionnaire |
| Reasons for Non-adherence (Patient-related Factors) | | |
|  | Over the past 4 wk I have never felt blue, downhearted, or depressed. | Reduced GTCAT |
|  | My overall health is excellent. | Reduced GTCAT |
|  | Does taking your medication(s) affect the way you live your life? | Medication Adherence Survey for Hemodialysis Patients |
|  | Do you remember to take your medication(s)? | Medication Adherence Survey for Hemodialysis Patients |
|  | Do you think you are taking too many medication(s)? | Medication Adherence Survey for Hemodialysis Patients |
|  | Do you have your own way to remember to take your medication(s)? | Medication Adherence Survey for Hemodialysis Patients |
|  | Do you feel that you need to know more about what your medication(s) are for? | Medication Adherence Survey for Hemodialysis Patients |
|  | Does someone (spouse/family/friends) help you remember to take your medication(s)? | Medication Adherence Survey for Hemodialysis Patients |
|  | Does taking your medication(s) make you feel upset? | Medication Adherence Survey for Hemodialysis Patients |
|  | Do you change the way you take any of your medication(s) from what the doctor/nurse/pharmacist recommended? | Medication Adherence Survey for Hemodialysis Patients |
|  | If you change the way you take your medication(s) what do you do differently? Please select all that apply to you: ˆ I change how many times a day I take them. ˆ I skip some of the doses. ˆ I stop taking the medication(s). ˆ I do not take the medication(s) with/without food like the doctor/nurse/pharmacist suggested because they are too hard to take with/without food. ˆ I find some of the medication(s) too hard to take at the same time as others like the doctor/nurse/pharmacist suggested. ˆ I change some of the medication(s) that were prescribed to something I feel works the same. ˆ I do not change the way I take my medication(s). | Medication Adherence Survey for Hemodialysis Patients |
|  | Why do you sometimes need to change the way you take your medication(s)? Please select all that apply to you: ˆ There are too many medication(s).  ˆ I do not feel I need all of these medication(s). ˆ I only take medication(s) when I feel ill. ˆ I have a hard time swallowing some or all of my medication(s) ˆ I cannot get all of my medication(s) from the pharmacy. ˆ I only take medication(s) when my labs are not normal. ˆ My medication(s) are too expensive. ˆ I have a hard time remembering when to take my medication(s). ˆ I am worried about my medications interacting with each other. ˆ I do not like the taste/smell/look of my medication(s). ˆ I do not understand what the medications are for. ˆ I sometimes need to take a break from my medication(s). ˆ I do not change the way I take my medication(s). ˆ I am not able to read/understand the directions for taking my medication(s). ˆ I do not feel the medication(s) result in a visible health benefit. | Medication Adherence Survey for Hemodialysis Patients |
|  | Medication can help alleviate my symptoms. | BMAS |
|  | Taking medication is a burden to me. | BMAS |
|  | I need to take medication regularly. | BMAS |
|  | By staying on medication, I can prevent getting sick and going back to the hospital. | BMAS |
|  | I follow the doctor's instruction to take medication. | BMAS |
|  | I forget to take my medication. | BMAS |
|  | I adjust the medication and dosage based on my condition. | BMAS |
|  | If I have the side effect of the medication, I will stop taking it. | BMAS |
|  | I take medication only when I am sick. | BMAS |
|  | Over the past 7 days, I missed my dose because I was out of my routine. | DOSE-Nonadherence |
|  | Over the past 7 days, I missed my dose because the medication caused side effects. | DOSE-Nonadherence |
|  | Over the past 7 days, I missed my dose because I could not afford the medication. | DOSE-Nonadherence |
|  | Over the past 7 days, I missed my dose because the medication affected my sex life. | DOSE-Nonadherence |
|  | Over the past 7 days, I missed my dose because treatment was hard on my family. | DOSE-Nonadherence |
|  | Over the past 7 days, I missed my dose because I had other medications to take. | DOSE-Nonadherence |
|  | Over the past 7 days, I missed my dose because I was afraid the medication would interact with other medication I take, | DOSE-Nonadherence |
|  | Over the past 7 days, I missed my dose because I was feeling too sick to take it. | DOSE-Nonadherence |
|  | Over the past 7 days, I missed my dose because I forgot. | DOSE-Nonadherence |
|  | Over the past 7 days, I missed my dose because I did not have my medicines with me. | DOSE-Nonadherence |
|  | Over the past 7 days, I missed my dose because I was too late with my dose. | DOSE-Nonadherence |
|  | Over the past 7 days, I missed my dose because I was asleep. | DOSE-Nonadherence |
|  | Over the past 7 days, I missed my dose because there was no one to help me. | DOSE-Nonadherence |
|  | Over the past 7 days, I missed my dose because I ran out of medication. | DOSE-Nonadherence |
|  | Over the past 7 days, I missed my dose because I could not meet the food requirements. | DOSE-Nonadherence |
|  | Over the past 7 days, I missed my dose because the medication was not working. | DOSE-Nonadherence |
|  | Over the past 7 days, I missed my dose because I did not want others to see my medications. | DOSE-Nonadherence |
|  | Over the past 7 days, I missed my dose because I could not get answers to my questions about the medication. | DOSE-Nonadherence |
|  | Do you know the name of your medications? | Self-Assessment Tool to Measure Imatinib Adherence in Patients with Chronic Myeloid Leukemia |
|  | Does a change in your daily routine modify the way you take your medicine? | Self-Assessment Tool to Measure Imatinib Adherence in Patients with Chronic Myeloid Leukemia |
|  | Sometimes if you feel worse when you take your medicine, do you stop taking it? | Self-Assessment Tool to Measure Imatinib Adherence in Patients with Chronic Myeloid Leukemia |
|  | Do you think that you take too many medications? | Self-Assessment Tool to Measure Imatinib Adherence in Patients with Chronic Myeloid Leukemia |
|  | Do you ever not take your medicine because you forgot to do so? | Self-Assessment Tool to Measure Imatinib Adherence in Patients with Chronic Myeloid Leukemia |
|  | Do you ever miss doses of your medicine when you feel sick? | Self-Assessment Tool to Measure Imatinib Adherence in Patients with Chronic Myeloid Leukemia |
|  | Do you sometimes skip doses of your medicine when you feel better? | Self-Assessment Tool to Measure Imatinib Adherence in Patients with Chronic Myeloid Leukemia |
|  | I visit the doctor with the recommended frequency | Assessment Scale for Treatment Compliance in Type 2 Diabetes Mellitus |
|  | I feel strong enough to fight the disease. | Assessment Scale for Treatment Compliance in Type 2 Diabetes Mellitus |
|  | I feel anxious when it is medication/insulin time. | Assessment Scale for Treatment Compliance in Type 2 Diabetes Mellitus |
|  | I believe that my disease will completely cure when my worries or stress is over. | Assessment Scale for Treatment Compliance in Type 2 Diabetes Mellitus |
|  | I am angry because I have to eat special food and have special needs. | Assessment Scale for Treatment Compliance in Type 2 Diabetes Mellitus |
|  | I always feel depressed about my future due to my disease. | Assessment Scale for Treatment Compliance in Type 2 Diabetes Mellitus |
|  | I do not trust health staff; they do not help me. | Assessment Scale for Treatment Compliance in Type 2 Diabetes Mellitus |
|  | I use a reminder to help me take my medications. | Patient-Reported Measures Assessing Adherence Behaviors and Barriers in Patients Living with HIV |
|  | I have a routine that works well for taking my medication as prescribed. | Patient-Reported Measures Assessing Adherence Behaviors and Barriers in Patients Living with HIV |
|  | I keep my medications in a certain place to help me remember to take them. | Patient-Reported Measures Assessing Adherence Behaviors and Barriers in Patients Living with HIV |
|  | In the past 30 days, I skipped taking some or all of my medications ON PURPOSE. | Patient-Reported Measures Assessing Adherence Behaviors and Barriers in Patients Living with HIV |
|  | What the doctor tells me, I hang on to. | CQR5 |
|  | During the last four weeks, how many times were you careless about taking your medication? | AAS |
|  | During the last four weeks, how many times, when you felt better, did you stop taking your medication? | AAS |
|  | During the last four weeks, how many times, when you felt worse, did stop taking your medication? | AAS |
|  | How often do you decide not to take your medicine? | ARMS |
|  | How often do you miss taking your medicine when you are careless? | ARMS |
|  | How often do you change the dose of your medicines to suit your needs (like when you take more or less pill than you’re supposed to)? | ARMS |
|  | How often do you forget to get prescriptions filled? | ARMS |
|  | How often do you run out of medicine? | ARMS |
|  | How often do you plan ahead and refill your medicines before they run out? | ARMS |
|  | How often do you forget to take your medicine when you are supposed to take it more than once a day? | ARMS |
|  | How often do you forget to take your medicine? | ARMS |
|  | How often do you miss taking your medicine when you feel better? | ARMS |
|  | How often do you miss taking your medicine when you feel sick? | ARMS |
|  | You have been prescribed medication(s) for your health condition(s) which is to be taken regularly. How would you describe your past experience with taking your medication(s)?  a. I want to be very regular in taking my medication(s), but I am not always good with it due to some challenges.  b. I take my medication(s) regularly (9 out of 10 times).  c. I am not very regular in taking my medication(s) because I feel unwilling. | M-DRAW |
|  | Do you feel that you can take more or less of your medication than the prescribed dose to fit your lifestyle? | M-DRAW |
|  | Do you consider it a burden that you have to take your medications for the rest of your life? | M-DRAW |
|  | Do you have doubts about whether your health condition needs to be treated? | M-DRAW |
|  | Do you have doubts if taking your medication will improve your health condition in the long term? | M-DRAW |
|  | Do you have any other doubts or concerns about taking your medication? | M-DRAW |
|  | Do you feel unsure about how/when to take your medications? | M-DRAW |
|  | Do you forget to place refill requests on time? | M-DRAW |
|  | Do you feel like you don’t get any benefits from taking your medication? | M-DRAW |
|  | Do you feel that you are NOT receiving the best possible treatment available from your health care provider? | M-DRAW |
|  | Do you worry about what foods or other medications might interact with your medication? | M-DRAW |
|  | Do you feel uncomfortable about taking your medication while you are out with family and friends? | M-DRAW |
|  | It bothers me that others know that I take this medication. | AMBAS |
|  | I'm ashamed of taking this medication. | AMBAS |
|  | It's the same whether I take this medication or not. | AMBAS |
|  | When I take this medication it's like I'm not myself. | AMBAS |
|  | I find it difficult to take the medication as recommended by the doctor (which pills, at what time…). | AMBAS |
|  | It bothers me when my medication is changed. | AMBAS |
|  | I plan to stop this medication when feeling better. | AMBAS |
|  | My medication has more positive effects than negative ones. | AMBAS |
|  | When I take this medication, I can think in a clearer manner. | AMBAS |
|  | In the last month, did you take your medication(s) with you when you are away from home (e.g., traveling or visiting relatives)? | IADMAS |
|  | During sick days (e.g., flu, and diarrhea), did you take less of your medication (s) without consulting a physician due to reduced appetite? | IADMAS |
|  | In the last month, how often did you intend to take your medication(s) in doses different to what has been prescribed? | IADMAS |
|  | In the last month, how often did you intend to take your medication(s) in a time different to what has been prescribed? | IADMAS |
|  | In the last month, did you take less of your medication (s) without consulting a physician because you feel better? | IADMAS |
|  | Do you have difficulty in remembering to take your medications? | GMAS |
|  | Do you forget to take your medication due to your busy schedule, travelling, meeting, events at home, party, marriage, religious celebrations, etc.? | GMAS |
|  | Do you stop taking medications without informing the doctor? | GMAS |
|  | Do you discontinue your medicines due to other medicines that you have to take for your additional disease? | GMAS |
|  | Do you find it is a hassle to remember your medications due to medication regime complexity? | GMAS |
|  | During the last month, had there been any occasion when you missed your medicines due to progression of disease and addition of new medicines? | GMAS |
|  | Do you alter medication regimen, dose and frequency by yourself? | GMAS |
|  | Do you discontinue these medications because they are not worth of the money you spent on them? | GMAS |
|  | Do you know the long-term prognosis of your disease? | Chinese and Western Medication Adherence Scale in Chronic Kidney Disease |
|  | Do you know the names and usage of your medicines? | Chinese and Western Medication Adherence Scale in Chronic Kidney Disease |
|  | Do you know what symptoms will develop when you get worse? | Chinese and Western Medication Adherence Scale in Chronic Kidney Disease |
|  | Do you know the primary role of your medicines? | Chinese and Western Medication Adherence Scale in Chronic Kidney Disease |
|  | I personally search for and collect information that I want about my medicine. | 12-Item Medication Adherence Scale for Patients with Chronic Disease |
|  | I accept the necessity of taking medication in the prescribed manner to treat my illness. | 12-Item Medication Adherence Scale for Patients with Chronic Disease |
|  | Taking medication is part of my everyday life, just like eating or brushing my teeth. | 12-Item Medication Adherence Scale for Patients with Chronic Disease |
|  | I have stopped taking medication based on my own judgment (not including times when I forgot to take my medication) | 12-Item Medication Adherence Scale for Patients with Chronic Disease |
|  | I report side effects, allergic reactions, or unusual symptoms caused by the medication | 12-Item Medication Adherence Scale for Patients with Chronic Disease |
|  | I sometimes get annoyed that I have to keep taking medicine every day. | 12-Item Medication Adherence Scale for Patients with Chronic Disease |
|  | Do you know why you are taking the medicines? | ICAMP |
|  | Some families tell us that their child worries them or makes it difficult to give them the medicines. Have you not taken medicines for any of these reasons:  □ I do not know why taking am taking the medicines or keeps asking questions about the medicines  □ I forgot to take medicine □ I felt ill or was vomiting  □ I was playing or at school or work  □ I refused to take medicine  □ I have problems with 1 formulation (tablets, liquids)  □ I find medicines too bitter □ I can’t take without food □ None of the above □ Other (specify) | ICAMP |
|  | Taking pills everyday is not a big deal. | Lasso-10 |
|  | In the past month, have you missed taking your medications because you have to wake up very early to go to work and no time to eat? | IRT-30 |
|  | In the past month, have you missed taking your medications because you didn’t want to bring my pills to social activities (restaurant, friend’s home)? | IRT-30 |
|  | In the past month, have you missed taking your medications because you wanted to have a free day without pills? | IRT-30 |
|  | In the past month, have you missed taking your medications because you forgot? | IRT-30 |
|  | In the past month, have you missed taking your medications because you were busy doing other things (e.g., working, trying to survive, getting food?)? | IRT-30 |
|  | In the past month, have you missed taking your medications because you were too busy at work, school, or home? | IRT-30 |
|  | In the past month, have you missed taking your medications because you lost track of time? | IRT-30 |
|  | In the past month, have you missed taking your medications because you didn’t have a good night sleep? | IRT-30 |
|  | I skipped a dose of my medication because I was worried about its side effects. | MEDS |
|  | I took a smaller amount of my medication because I was worried about its side effects. | MEDS |
|  | I skipped a dose of my medication because I was worried about getting addicted to it. | MEDS |
|  | I took a smaller amount of my medication because I was worried about getting addicted to it. | MEDS |
|  | I skipped a dose of my medication because I did not need it. | MEDS |
|  | I took a smaller amount of my medication because I did not need it. | MEDS |
|  | Do you think your treatment will last too long? | Risk of Nonadherence to Antibiotic Treatment Questionnaire |
|  | Do you just forget to take medicines some of the time? | Risk of Nonadherence to Antibiotic Treatment Questionnaire |
|  | Have you taken a medicine more or less often than prescribed? | Risk of Nonadherence to Antibiotic Treatment Questionnaire |
|  | Have you skipped or stopped taking a medicine because you did not think it was working? | Risk of Nonadherence to Antibiotic Treatment Questionnaire |
|  | Are you careless at times about taking your medicine? | Risk of Nonadherence to Antibiotic Treatment Questionnaire |

Abbreviations:

Antidepressant Adherence Scale (AAS); Antipsychotic Medication Beliefs and Attitudes Scale (AMBAS); Adherence to Refills and Medications Scale (ARMS); Brief Medication Adherence Scale (BMAS); 5-Item Compliance Questionnaire for Rheumatology (CQR5); Diagnostic Adherence to Medication Scale (DAMS); Domains of Subjective Extent of Nonadherence (DOSE-Nonadherence); General Medication Adherence Scale (GMAS); Reduced Glaucoma Treatment Compliance Assessment Tool (GTCAT); HIV: Human immunodeficiency virus; Iraqi Anti-Diabetic Medication Adherence Scale (IADMAS); Modified Drug Adherence Work-Up (M-DRAW); Medication Adherence Estimation and Differentiation Scale (MEDS); Multiple Sclerosis Treatment Adherence Questionnaire (MS-TAQ); Simplified Medication Adherence Questionnaire (SMAQ); Treatment Adherence Survey – Patient Version (TAS-P)
